# Revisiting the bromination of 3β-hydroxycholest-5-ene with CBr_4_/PPh_3_ and the subsequent azidolysis of the resulting bromide, disparity in stereochemical behavior

**DOI:** 10.3762/bjoc.19.9

**Published:** 2023-01-27

**Authors:** Christian Schumacher, Jas S Ward, Kari Rissanen, Carsten Bolm, Mohamed Ramadan El Sayed Aly

**Affiliations:** 1 Institute of Organic Chemistry, RWTH Aachen University, Landoltweg 1, 52074 Aachen, Germany; 2 University of Jyvaskyla, Department of Chemistry, P.O. Box 35, 40014 Jyväskylä, Finland; 3 Chemistry Department, Faculty of Science, Port Said University, 42522-Port Said, Egypt

**Keywords:** Appel reaction, azidolysis, cholesterol, crystal structure, Walden inversion

## Abstract

Cholesterol reacts under Appel conditions (CBr_4_/PPh_3_) to give 3,5-cholestadiene (elimination) and 3β-bromocholest-5-ene (substitution with retention of configuration). Thus, the bromination of cholesterol deviates from the stereochemistry of the standard Appel mechanism due to participation of the Δ^5^ π-electrons. In contrast, the subsequent azidolysis (NaN_3_/DMF) of 3β-bromocholest-5-ene proceeds predominantly by Walden inversion (S_N_2) affording 3α-azidocholest-5-ene. The structures of all relevant products were revealed by X-ray single crystal structure analyses, and the NMR data are in agreement to the reported ones. In light of these findings, we herein correct the previous stereochemical assignments reported by one of us in the *Beilstein J. Org. Chem.* **2015**, *11*, 1922–1932 and the *Monatsh. Chem.* **2018**, *149*, 505–517.

## Introduction

3β-Hydroxycholest-5-ene (cholesterol) is a structural and physiologic amphipathic steroid in human and animals as well. Cholesterol is an essential component of the plasma membrane, where it acts as fluidity buffer, permeability switch, and consequently in cell signaling pathways. Physiologically, cholesterol is the substrate for the biosynthesis of steroidal hormones, vitamin D and bile acids [1–2].

**Figure 1:**
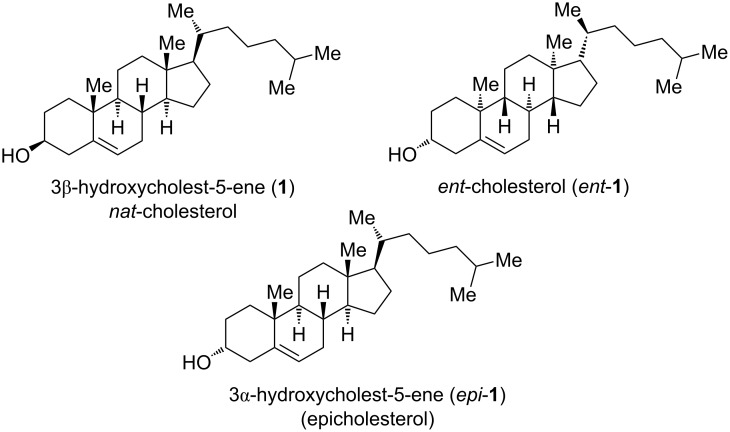
Chemical structure of three isomeric cholesterols.

Although cholesterol can adopt 256 stereoisomeric structures, biological significances were only reported for the natural compound (*nat*-cholesterol, **1**) and its enantiomer (*ent*-cholesterol, *ent*-**1**) (Figure 1) [3]. While **1** and *ent*-**1** are characterized by hydroxy groups in β-position at C3, epicholesterol (*epi*-**1**) has an α-OH at C3.

Diets of animal sources like red meat, liver, milk, and butter provide the body with its daily needs of cholesterol. In addition, hepatocyctes synthesize cholesterol through the mevalonate pathway. Dietary cholesterol is absorbed into the blood stream through a specific membrane bound protein named Niemann-Pick C1-Like 1 (NPC1L1) on the gastrointestinal tract epithelial cells as well as in hepatocytes. As hydrophobic molecule, it circulates in the blood stream engulfed in carrier lipoproteins of two types, high density lipoproteins (HDL) or good proteins and low-density lipoproteins (LDL) or bad proteins [4–5].

People with a total blood cholesterol over 125–200 mg/dL are considered hypercholesterimic. They are under high risk of cholelithiasis (formation of gallstones), atherosclerosis, heart attack, stroke, peripheral artery disease, and cancer [4–5]. Synergistic cholesterol lowering medications are inhibitors of cholesterol absorption (ezetimibe) and cholesterol biosynthesis (statins). However, the side effects of these drugs are controversial. Therefore, synthetic cholesterol derivatives came into focus for recent applications in chemical biology and materials science [6]. The advances have been summarized in comprehensive reviews [7–8].

**Figure 2:**
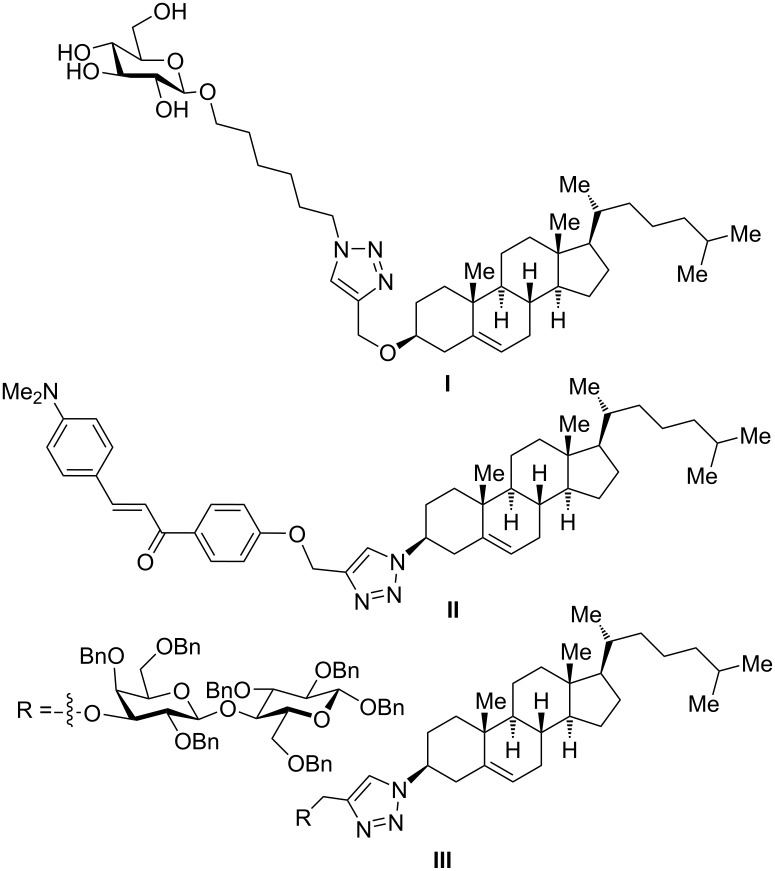
Selected previously described cholesterol derivatives with interesting antibacterial and cytotoxic activities [9–10]. The current results correct the structure of cholesterols **II** and **III** to be in the α- and not, as reported before and shown in this Figure, in the β-configuration.

In previous studies, one of us (M. R. E. A.) felt intrigued by the potential of chemical hybridization of cholesterol through simple connections of pharmacophores including sugars, chalcones, quinolone, theophylline, and ferrocene using click chemistry [9–11]. Following this strategy, cholesterol was propargylated, coupled with azido quinoline, and then functionalized with glucose as part of random designs to discover new antimicrobial and cytotoxic candidates. From these studies, conjugates **I** [9] and **II** [10] were identified to display an excellent preliminary antibacterial impact, and congener **III** [10] showed a good cytotoxic effect against the prostate cancer cell line PC-3 (Figure 2). When the spacer of **I** was increased from C_6_ to C_11_, the antimicrobial potential dramatically decreased [11].

In order to extend the compound platform, the synthesis of 3-azidocholest-5-ene was addressed [10]. Starting from natural cholesterol, a double inversion of the stereogenic centre at C3 through an Appel type two step conversion of cholesterol into the 3-azido derivative via the corresponding bromide was assumed. Thus, the expected product was 3β*-*azidocholest-5-ene [10]. Lacking crystallographic evidence, the synthetic chemistry was expanded to click conjugates such as **II** and **III**, and the data was reported [10].

Recently, those studies were revisited, and we now obtained single crystals which allowed to unequivocally establishing the relative configurations of the products by X-ray crystallography. Accordingly, the stereochemistry at C3 of the bromo-, azido- and triazolocholesterols was incorrectly assigned, and we now wish to correct the previous reported structures.

## Results and Discussion

3β-Hydroxy-Δ^5^-steroids, for instance, cholesterol, pregnenolone, and their derivatives which possess a potential leaving group at the 3β-position, have a unique feature in their chemical reactions. In these steroids, the breaking of the C3–X bond is facilitated by the formation of a cationic strained cyclopropane intermediate, which is formed by translocation of the C5–π bond electrons to the homoallylic carbon atom at C3 [12]. In this way, substitutions at the stereogenic homoallylic carbon atom can proceed with retention of configuration. Concurrently, a so-called *i*-steroid rearrangement leads, for instance, to 6β-azido-3α,5-cyclo-5α-cholestane by 6β-face attack of the steroidal substrate by the nucleophile [13–14].

**Scheme 1:**
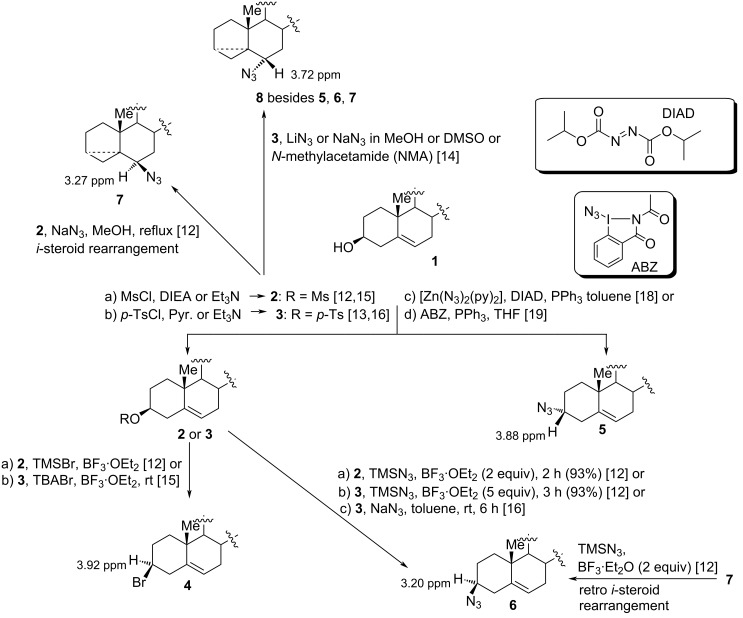
Stereochemical outcome of OH/N_3_ transformations under different conditions.

In similar work, Peterson and co-workers reported several examples of such stereoretentive conversions of cholesterol, which provided the corresponding 3β-halo- and 3β-azido-5-cholesterenes in high yields [12]. The cholesterol mesylate was the most effective intermediate, and the nucleophiles were trimethylsilyl-based nucleophiles. TiCl_4_ and BF_3_·OEt_2_ served as activators. No reaction was observed with the 3*α*-mesyl analog and the cholestane congener. The 3β-azido derivative could also be obtained from 6β-azido-3α,5-cyclo-5α-cholestane [14] by treatment with a mixture of TMSN_3_ and BF_3_·OEt_2_ [12]. All of those results confirmed the involvement of regio- and stereospecific *i*-steroid and retro*-i*-steroid rearrangements. Later, tetrabutylammonium halides were used as cost effective and stable alternatives of TMS-based reagents [15]. Treatment of compound **4** (Scheme 1) with NaN_3_ in refluxing toluene was reported to proceed with retention of configuration to afford the β-epimer **6** [16]. Another nice application of this chemistry was recently reported by Oestreich and co-workers, who converted 3β-hydroxypregn-5-en-20-one into the corresponding 3-bromo derivative, which also occurred with retention of configuration at C3 [17].

In 2008, a direct dehydroxyazidation of cholesterol by treatment of the steroid with a zinc azide–pyridine complex, diisopropyl azodicarboxylate (DIAD), and PPh_3_ was described [18]. This Mitsunobu-like reaction occurred with complete inversion at C3 to afford 3α-azidocholest-5-ene (**5**) in high yield. The same product was recently obtained by direct dehydroxyazidation of cholesterol upon treatment with *N*-acetyl azidobenziodazolone (ABZ) and PPh_3_ in THF [19], Table 1.

**Table 1:** Manipulations for bromination and azidation of cholesterol.

Entry	C3 Arm	Reagent	Catalyst	Solvent	Product	Ref.

1	β-OH	Zn(N_3_)(py)_2_	DIAD/PPh_3_	toluene	**5**	[18]
2	β-OH	ABZ	PPh_3_	THF	**5**	[19]
3	β-OMs	TMSBr	BF_3_·OEt_2_	DCM	**4**	[12]
4	β-OMs	TBABr	BF_3_·OEt_2_	DCM	**4**	[15]
5	β-OMs	TMSN_3_	BF_3_·OEt_2_	DCM	**6**	[12]
6	β-OTs	TMSN_3_	BF_3_·OEt_2_	DCM	**6**	[12]
7	β-OMs	NaN_3_	–	MeOH	**7**	[12]
8	β-OTs	NaN_3_	–	toluene	**6**	[16]
9	β-OTs	LiN_3_ or NaN_3_	–	MeOH or DMSO or NMA	**5**–**8**	[14]

**Scheme 2:**
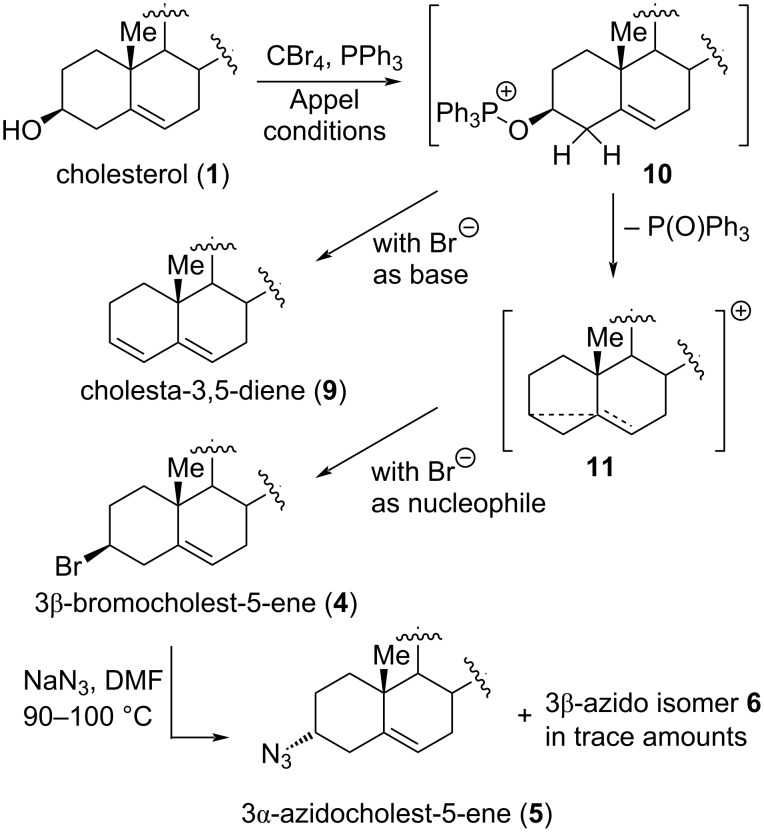
Mechanistic interpretation of the conversion of cholesterol **1** into diene **9**, bromide **4**, and azides **5** and **6**.

While synthesizing new potential biologically active probes with cholesterol scaffolds in the Port Said laboratories, particularly from 3-azidocholest-5-ene, we started wondering about the previously reported structural and stereochemical assignments of the steroid derivatives. After repeating the C–OH to bromide exchange of cholesterol (**1**) under Appel conditions, we now found two products in different yields, **4** (80%), and **9** (8%), Scheme 2. Their polarities were so similar that they merged during color development on the hot TLC plate. Compounds **4** and **9** displayed in petroleum ether *R*_f_ values of 0.75 and 0.78, respectively. Finally, the two compounds could be separated by flash chromatography on silica gel of different mesh numbers. Single crystals of both were obtained by slow evaporation from diethyl ether.

**Figure 3:**
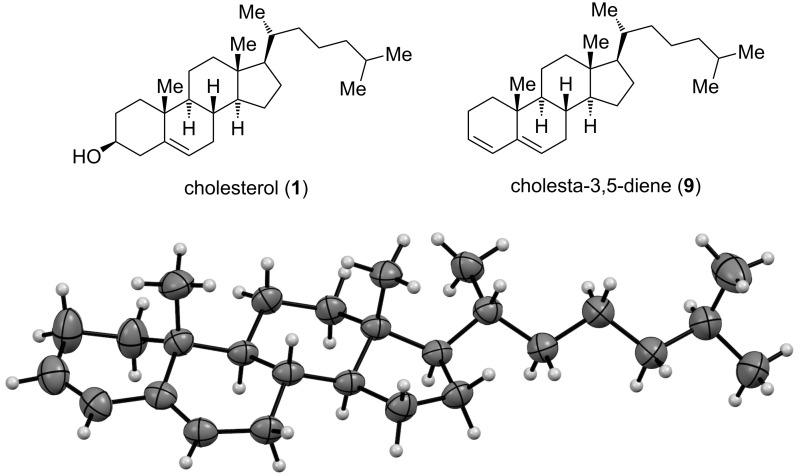
Top: cholesterol (**1**) and the less polar product from the Appel reaction, cholesta-3,5-diene (**9**); bottom: X-ray structure of **9** with thermal displacement parameter at 50% probability level.

The less polar, minor material gave ice-white needles, and an X-ray single crystal structure determination revealed the product to be cholesta-3,5-diene (**9**, Figure 3).

The ^1^H NMR data of **9** are in full agreement with those reported by others [20–22]. This compound was erroneously assigned as 3*α*-bromocholest-5-ene [10].

**Figure 4:**
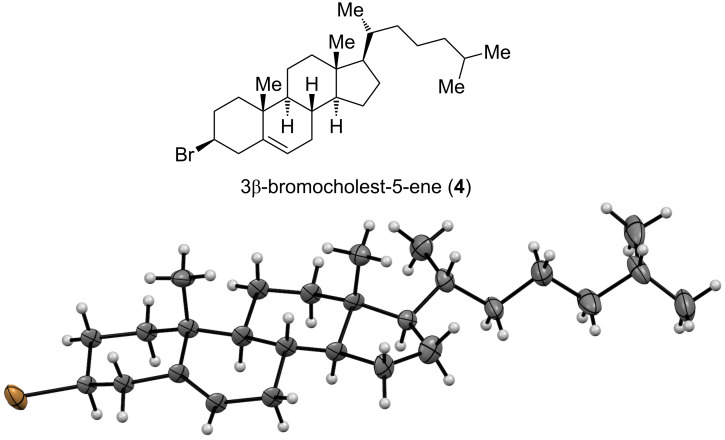
Top: the more polar product from the Appel reaction 3β-bromocholest-5-ene (**4**); bottom: X-ray structure of **4** with thermal displacement parameter at 50% probability level.

The major, slightly more polar product of the Appel reaction was 3β-bromocholest-5-ene (**4**, Figure 4). It crystallized as colorless plates, and the structural and stereochemical assignment of **4** was unequivocally confirmed by X-ray crystal structure determination. Compound **4** was erroneously reported to have the α-configuration at C3 [10]. Now, the NMR chemical shift data of **4** are in full agreement with those reported earlier [12].

**Figure 5:**
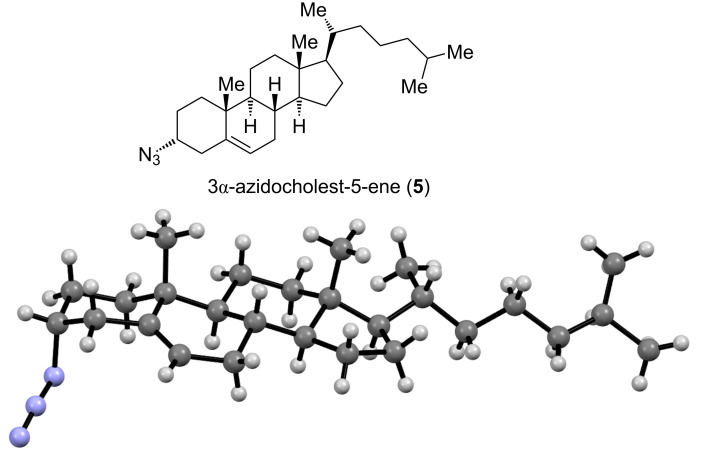
Top: 3α-azidocholest-5-ene (**5**) obtained by treatment of **4** with NaN_3_ in DMF; bottom: X-ray crystal structure [18] of **5** (CSD [24–25] refcode HOFFUE).

The reaction of 3-bromocholest-5-ene with NaN_3_ in DMF at 90–100 °C was reported to give 3β-azidocholest-5-ene [10]. Comparing the respective NMR data with the published ones [12] revealed a difference of about 0.6 ppm for the chemical shift of H3 suggesting a miss-assignment [23]. This assumption was confirmed by the X-ray structure analysis of single crystals obtained from Et_2_O, which showed the product to be 3α-azidocholest-5-ene (**5**, Figure 5) [18–19]. Thus, under the aforementioned conditions, 3β-bromocholest-5-ene (**4**) was predominantly converted into 3α-azidocholest-5-ene (**5**) involving a stereospecific transformation at C3 proceeding with a Walden inversion. For note, the ^1^H NMR spectrum of **5** revealed the presence of ca. 15% of the β-epimer **6**, which could result from an incomplete stereospecificity of the substitution opening an alternative reaction path. Also in this case, the NMR data are then in agreement with the reported ones [18].

In light of these results, the mechanistic interpretation depicted in Scheme 2 can be provided. Under Appel conditions with a combination of CBr_4_ and PPh_3_, 3β-hydroxycholest-5-ene (**1**) leads to two products, cholesta-3,5-diene (**9**) and 3β-bromocholest-5-ene (**4**). Both **9** and **4** result from intermediate **10**, in which the C3 hydroxy of **1** is activated. Deprotonation of **10** at C2 with bromide as base provides diene **9** as the minor product. Bromide **4** is formed via cyclopropyl cation **11**, which is generated from **10** by loss of triphenylphosphine oxide being supported by involvement of the Δ^5^ π-bond electrons from the α-face. Stereospecific reaction of **11** with bromide as nucleophile leads to **4**, in which the halo substituent is located on the β-side of the molecule. Treatment of **4** with NaN_3_ in DMF at 90–100 °C provides predominantly azide **5** [23]. This reaction has a high stereospecificity as well, proceeding mostly with inversion of configuration at C3 (Walden inversion). Consequently, the newly introduced substituent is located on the α-face of the steroid. Interestingly, this result contrasts the one observed when 3β-mesylcholest-5-ene is treated with TMSN_3_/BF_3_·OEt_2_ [12]. There, the process proceeds by retention of configuration locating the azido substituent on the β-face of the steroid (compound **6**).

## Conclusion

**Figure 6:**
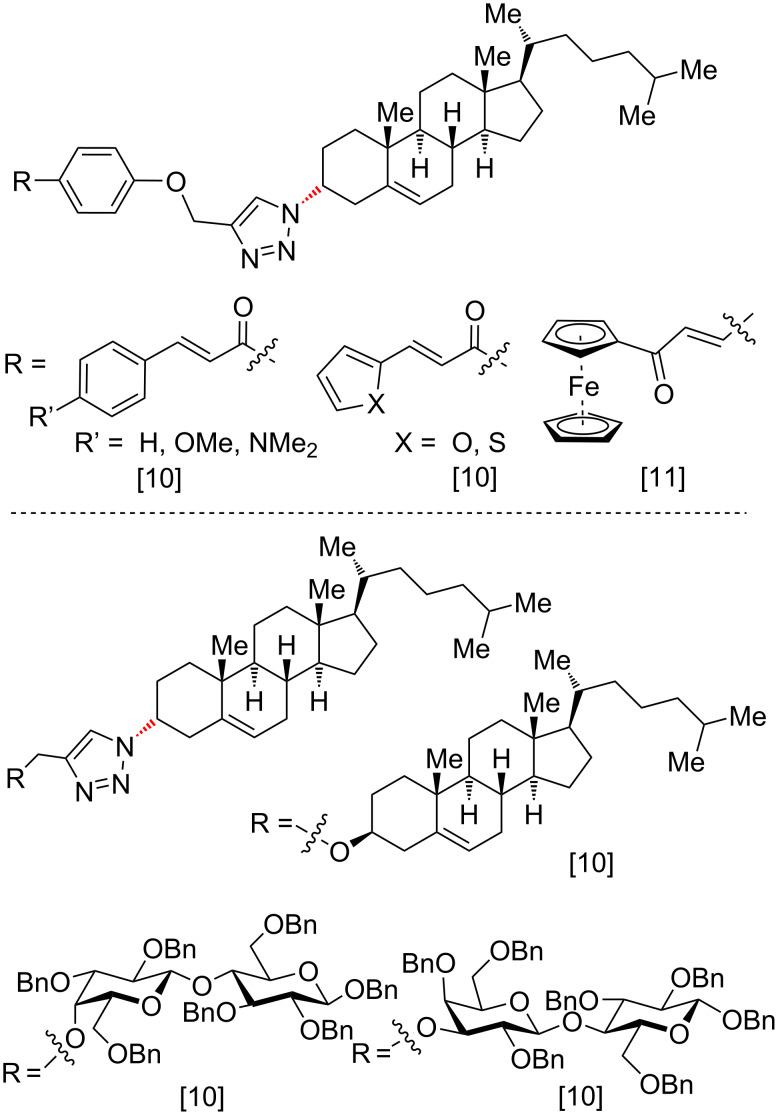
Compounds (next to **4**, **5** and **9**) to be corrected in refs. [10] and [11]. The respective bonds are highlighted in red.

For each product **4**, **5** and **9** the stereochemical assignment has now been confirmed by X-ray single crystal structure determination, and the NMR data are in agreement with those of previous reports. Former structural interpretations of **4**, **5** and **9** as well as those of follow-up compounds [10–11] need to be corrected as shown in (Figure 6).

## Experimental

### General information

Cholesterol was purchased from Advent/India, CBr_4_ was purchased from Sigma-Aldrich while, NaN_3_ and PPh_3_ were purchased from Across. Diethyl ether was purchased from Sigma-Aldrich, while petroleum ether (60–80 °C) and acetone were purchased from Oxford chemicals/India and DMF was purchased from Loba/India. All solvents were pure and used without further purification. Dichloromethane was purchased from Al Nasr/Egypt and dried over CaO before distillation. Flash chromatography was carried out on silica gel (Baker, 30–60 µm) (*Type-I* silica gel) and LiChroprep Si 60 (Merck; Ø (15–25 µm) (*Type-II* silica gel). TLC Monitoring tests were carried out using plastic sheets precoated with silica gel 60 F_245_ (layer thickness 0.2 mm) purchased from Merck. Spots were visualized by their fluorescence under UV–lamp (λ = 245 and 365 nm) or staining with iodine vapor or 15% H_2_SO_4_ or KMnO_4_ solution, or Ce(IV)SO_4_ in H_2_SO_4_. Melting points were determined on a Gallenkamp apparatus UK and are uncorrected. NMR spectra were recorded on a Bruker 600 MHz spectrometer at the central laboratory, King Abd El Aziz University, Jeddah, Saudi Arabia and a Bruker 400 Spectrometer at the Faculty of Pharmacy, Mansoura University, Mansoura, Egypt. The ^13^C NMR spectra are proton decoupled. IR spectra were recorded on a ATR–Alpha FT–IR Spectrophotometer 400–4000 cm^−1^ at Taif University, Taif, Saudi Arabia. Mass spectra were recorded on GCMS–QP 1000Ex Shimadzu spectrometers in the microanalysis unit at Cairo University, Cairo, Egypt.

### Reaction of 3β-hydroxycholest-5-ene (**1**) under Appel conditions

#### Cholest-3,5-diene (**9**) and 3β-bromocholest-5-ene (**4**)

As described in [10], a mixture of 3β-hydroxycholest-5-ene (**1**, 0.6 g, 1.5 mmol), PPh_3_ (0.5 g, 1.9 mmol) in DCM (5.0 mL) was stirred at ambient temperature, while CBr_4_ (0.6 g, 1.8 mmol) was added portionwise and stirring was continued for an hour. The mixture was evaporated in vacuo and the residue was subjected to flash chromatography on *Type-I* then *Type-II* silica gel (petroleum ether) to afford compound **9** (50.0 mg, 8.0%) as white sticks after recrystallization from Et_2_O and compound **4** (0.55 g, 80%) as creamy plates after recrystallization from Et_2_O.

Compound **9**: *R*_f_ = 0.78 (petroleum ether); mp: 92 °C^*^ (reported mp: 81.5–82.5 °C) [20]; ^1^H NMR (600 MHz, CDCl_3_) δ 5.70 (d, *J* = 9.6 Hz, 1H, H-4), 5.38–5.34 (m, 1H, H-3), 5.16 (m, 1H, H-6), 1.96–0.76 (m, 26H), 0.73 (s, 3H, CH_3_-19), 0.69 (d, 3H, *J*_20,21_ = 6.4 Hz, CH_3_-21), 0.65 (d, *J* = 1.2 Hz, 3H, CH_3_-26/CH_3_-27), 0.63 (d, *J* = 1.6 Hz, 3H, CH_3_-26/CH_3_-27), 0.48 (s, 3H, CH_3_-18); ^13^C {^1^H} NMR (150 MHz, CDCl_3_) δ 141.5, 129.0, 125.1, 123.2 (C-3, C-4, C-5, C-6), 57.0, 56.1, 48.4, 42.4, 39.8, 39.5, 36.2, 35.8, 35.2, 33.8, 31.78, 31.77, 28.2, 28.0, 24.2, 23.8, 23.0, 22.8, 22.6, 21.0, 18.8, 18.7 (22 carbons), 12.0 (CH_3_-18) [26]; C_27_H_44_ (368.34).

*This melting point was incorrectly attributed to 3α-bromocholest-5-ene in reference [10].

Compound **4**: *R*_f_ = 0.75 (petroleum ether); mp: 104 °C (reported mp: 99.5–100.5 °C) [12]; ^1^H NMR (400 MHz, CDCl_3_) δ 5.38 (dd, *J* = 2.5 Hz, 1H, H-6), 4.00–3.90 (m, 1H, H-3), 2.81–2.73 (m, 1H), 2.63–2.58 (m, 1H), 2.21–2.18 (m, 1H), 2.10–1.97 (m, 4H), 1.91–1.80 (m, 3H), 1.70–1.23 (m, 8H), 1.20–1.08 (m, 9H), 1.06 (s, 3H, CH_3_-19), 1.04–1.00 (m, 1H), 0.93 (d, *J*_20,21_ = 6.4 Hz, 3H, CH_3_-21), 0.90 (d, *J* = 1.4 Hz, 3H, CH_3_-26/CH_3_-27), 0.88 (d, *J* = 1.4 Hz, 3H, CH_3_-26/CH_3_-27), 0.70 (s, 3H, CH_3_-18); ^13^C{^1^H} NMR (100 MHz, CDCl_3_) δ 141.5 (C-5), 122.3 (C-6), 57.0, 56.1, 52.6, 50.2, 44.3, 42.3, 40.3, 39.8, 39.7, 36.4, 36.2, 35.8, 34.4, 31.8, 31.7, 28.2, 28.0, 24.3 (18 carbons), 23.8 (C-23), 22.9, 22.6 (C-26, C-27), 20.9 (C-11), 19.3 (C-19), 18.7 (C-21), 11.8 (CH_3_-18) [27]; EIMS (70 eV): calcd for C_27_H_45_Br: 448 [M]^+^; found: 449 (2) [M + H]^+^, 370 (3), 369 (17), 368 (53).

#### 3α-Azidocholest-5-ene (**5**)

As described in [10], a mixture of **4** (4.3 g, 9.5 mmol) and NaN_3_ (3.0 g, 46.1 mmol) in DMF (25 mL) was stirred at 90–100 °C for 48 h then diluted with H_2_O (25 mL). The mixture was extracted with dichloromethane (3 × 50 mL), dried over Na_2_SO_4_ and evaporated in vacuo. The residue was subjected to flash chromatography (petroleum ether) to afford compound **5** (2.47 g, 63%) as faint creamy sticks upon crystallization from Et_2_O. *R*_f_ = 0.26 (petroleum ether); mp: 98 °C [10] (reported mp: 114−115 °C [18]; IR: ν 2081 (N_3_ str) cm^−1^; ^1^H NMR (600 MHz, CDCl_3_) δ 5.39 (t, *J* = 2.3, 4.8 Hz, 1H, H-6), 3.87 (t, *J* = 3.0, 6.0 Hz, 1H, H-3), 3.20 (m, 0.15H),* 2.53 (ddd, *J* = 2.4, 2.4, 15.0 Hz, 1H, H-4a), 2.29 (d, *J* = 8.4 Hz, 0.3H),** 2.18 (ddd, *J* = 2.4, 2.4, 14.4 Hz, 1H, H-4b), 2.03–1.94 (m, 2H), 1.92–1.68 (m, 3H), 1.66–1.22 (m, 6H), 1.19–0.98 (m, 15H), 1.00 (s, 3H, CH_3_-19), 0.91 (d, *J*_20,21_ = 6.6 Hz, 3H, CH_3_-21), 0.87 (d, *J* = 2.4 Hz, 3H, CH_3_-26/CH_3_-27), 0.86 (d, *J* = 2.4 Hz, 3H, CH_3_-26/CH_3_-27), 0.68 (s, 3H, CH_3_-18); ^13^C{^1^H} NMR (150 MHz, CDCl_3_) δ 138.1 (C-5), 123.2 (C-6), 58.3 (C-3), 56.6 (C-14), 56.1 (C-17), 50.1 (C-9), 42.3 (C-13), 39.7 (C-12), 39.5 (C-24), 37.1 (C-10), 36.2 (C-22), 36.1 (C-4), 35.8 (C-20), 33.6 (C-1), 31.82, 31.8 (C-7, C-8), 28.2 (C-16), 28.0 (C-25), 26.1 (C-2), 24.1 (C-15), 23.8 (C-23), 22.8, 22.6 (C-26, C-27), 20.7 (C-11), 19.0 (C-19), 18.7 (C-21), 11.9 (C-18) [28]; EIMS (70 eV): calcd for C_27_H_45_N_3_: 411 [M]^+^; found: 412 (2) [M + H]^+^, 411 (4) [M]^+^, 393 (2), 383 (49), 368 (78).

*This signal is attributed to the H-3 of (15%) byproduct 3β-azidocholest-5-ene; **denotes to two protons of the 3β-azidocholest-5-ene epimer [12].

## Supporting Information

File 1 X-ray crystallography and NMR spectra.

File 2 Crystallographic information files for compounds **4** and **9**.
