# Supplementary material for: Revisiting the bromination of 3β-hydroxycholest-5-ene with CBr4/PPh3 and the subsequent azidolysis of the resulting bromide, disparity in stereochemical behavior
Source: Beilstein J Org Chem. 2023 Jan 27;19:91–9. doi: 10.3762/bjoc.19.9 (PMC9887750; doi:10.3762/bjoc.19.9)
Supplement: File 1 — X-ray crystallography and NMR spectra. [file Beilstein_J_Org_Chem-19-91-s001.pdf]

BEILSTEIN JOURNAL OF ORGANIC CHEMISTRY

Supporting Information

for

Revisiting the bromination of 3β-hydroxycholest-5-ene with CBr_4/PPh_3 and the subsequent azidolysis of the resulting bromide, disparity in stereochemical behavior

Christian Schumacher, Jas S. Ward, Kari Rissanen, Carsten Bolm
and Mohamed Ramadan El Sayed Aly

Beilstein J. Org. Chem. **2023,** *19,* 91–99. doi:10.3762/bjoc.19.9

X-ray crystallography and NMR spectra

License and Terms: This is a supporting information file under the terms of the Creative Commons Attribution License (https://creativecommons.org/licenses/by/4.0). Please note that the reuse, redistribution and reproduction in particular requires that the author(s) and source are credited and that individual graphics may be subject to special legal provisions.
The license is subject to the *Beilstein Journal of Organic Chemistry* terms and conditions: (https://www.beilstein-journals.org/bjoc/terms)

Table of Contents

I.	X-ray crystallography	S2
II.	NMR spectra	S4
III.	References	S26

I. X-ray crystallography

Due to the known stereochemistry of the cholesterol skeleton there is no need for ab initio absolute structure determination (viz. based on the Flack parameter). Single-crystal X-ray data for diene **9** was measured using a Bruker-Nonius Kappa CCD diffractometer with an APEX-II detector with graphite-monochromatized Mo-*K*α (λ = 0.71073 Å) radiation at 170 K. Data collection and reduction were performed using the program *COLLECT* [1] and *HKL DENZO AND SCALEPACK* [2], respectively, and the intensities were corrected for absorption using *SADABS* [3]. Single-crystal X-ray data for bromide **4** was measured using a Rigaku SuperNova dual-source Oxford diffractometer equipped with an Eos detector using mirror-monochromated Mo-*K*α (λ = 0.71073 Å) radiation at 120 K. The data collection and reduction were performed using the program *CrysAlisPro* and Gaussian face index absorption correction method was applied [4]. The structures were solved with intrinsic phasing (SHELXT) [5] and refined by full-matrix least squares on F^2 using the *OLEX2* software [6], which utilizes the *SHELXL* module [7]. For the azide **5** only a partial data collection was performed (see below) verifying the structure to be the known azide, 3α-azidocholest-5-ene [8].

Crystal data for the diene 9

$C_{27}H_{44}$, M = 368.62, colourless block, 0.24 × 0.30 × 0.40 mm, orthorhombic, space group $P2_12_12_1$, a = 7.5850(2) Å, b = 15.9238(4) Å, c = 19.4795(4) Å, V = 2352.77(10) Å^3, Z = 4, D_{calc} = 1.041 gcm^{-3}, F000 = 824, μ = 0.06 mm^{-1}, T = 170(1) K, θ_{max} = 28.7°, 5169 total reflections, 3861 with $I_o > 2\sigma(I_o)$, R_{int} = 0.052, 5169 data, 249 parameters, no restraints, GooF = 1.03, 0.28 < d$\Delta\rho$ < -0.17 eÅ^{-3}, $R[F^2 > 2\sigma(F^2)]$ = 0.053, $wR(F^2)$ = 0.131. CCDC-2204245.

Crystal data for the bromide 4

$C_{27}H_{45}Br$, M = 449.54, colourless plate, 0.06 × 0.17 × 0.25 mm^3, monoclinic, space group $P2_1$, a = 11.4127(12) Å, b = 7.5896(9) Å, c = 28.603(5) Å, β = 90.077(13)°, V = 2477.5(6) Å^3, Z = 4, D_{calc} = 1.205 gcm^{-3}, F000 = 968, μ = 1.67 mm^{-1}, T = 120.0(1) K, θ_{max} = 25.8°, 11562 total reflections, 5812 with $I_o > 2\sigma(I_o)$, R_{int} = 0.109, 11562 data, 467 parameters, 130 restraints, GooF = 1.06, 1.51 < d$\Delta\rho$ < -1.68 eÅ^{-3}, $R[F^2 > 2\sigma(F^2)]$ = 0.098, $wR(F^2)$ = 0.309. CCDC-2204246.

Verification of the structure of the azide 5

The physical habit (colorless plates), the unit cell (a = 13.2746(14) Å, b = 6.1854(6) Å, c = 14.9159(15) Å, β = 93.285(9)°, V = 1222.7(2) Å^3) and the space group ($P2_1$) matched that previously reported for 3α-azidocholest-5-ene [8]. A partial (30%) data set was collected and a figure base on it is shown in (Figure S1) confirming the studied sample to be the 3α-azidocholest-5-ene.

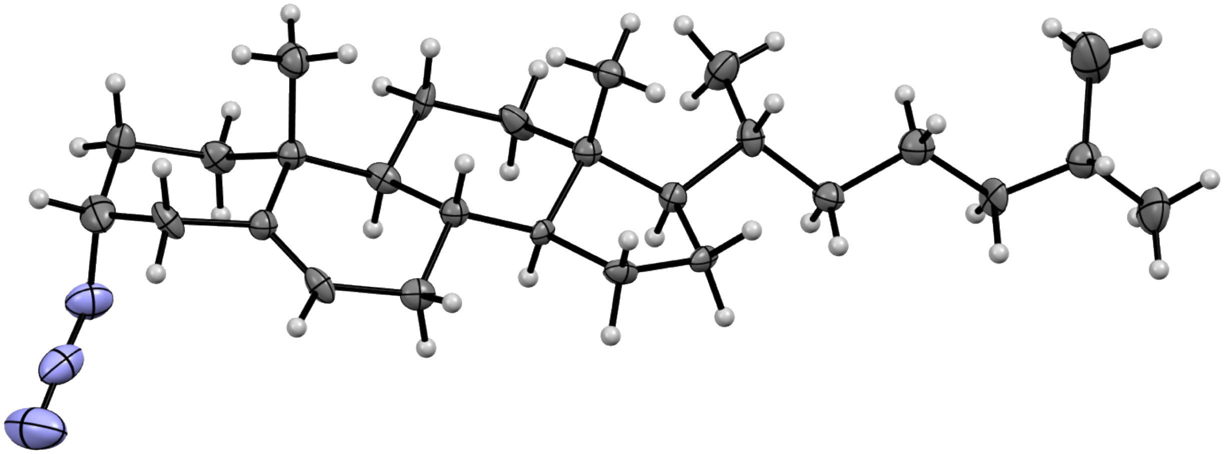

Fig. S1. The ORTEP plot of the 3α-azidocholest-5-ene from a partial data set with thermal displacement parameter at 50% probability level.

II. NMR spectra

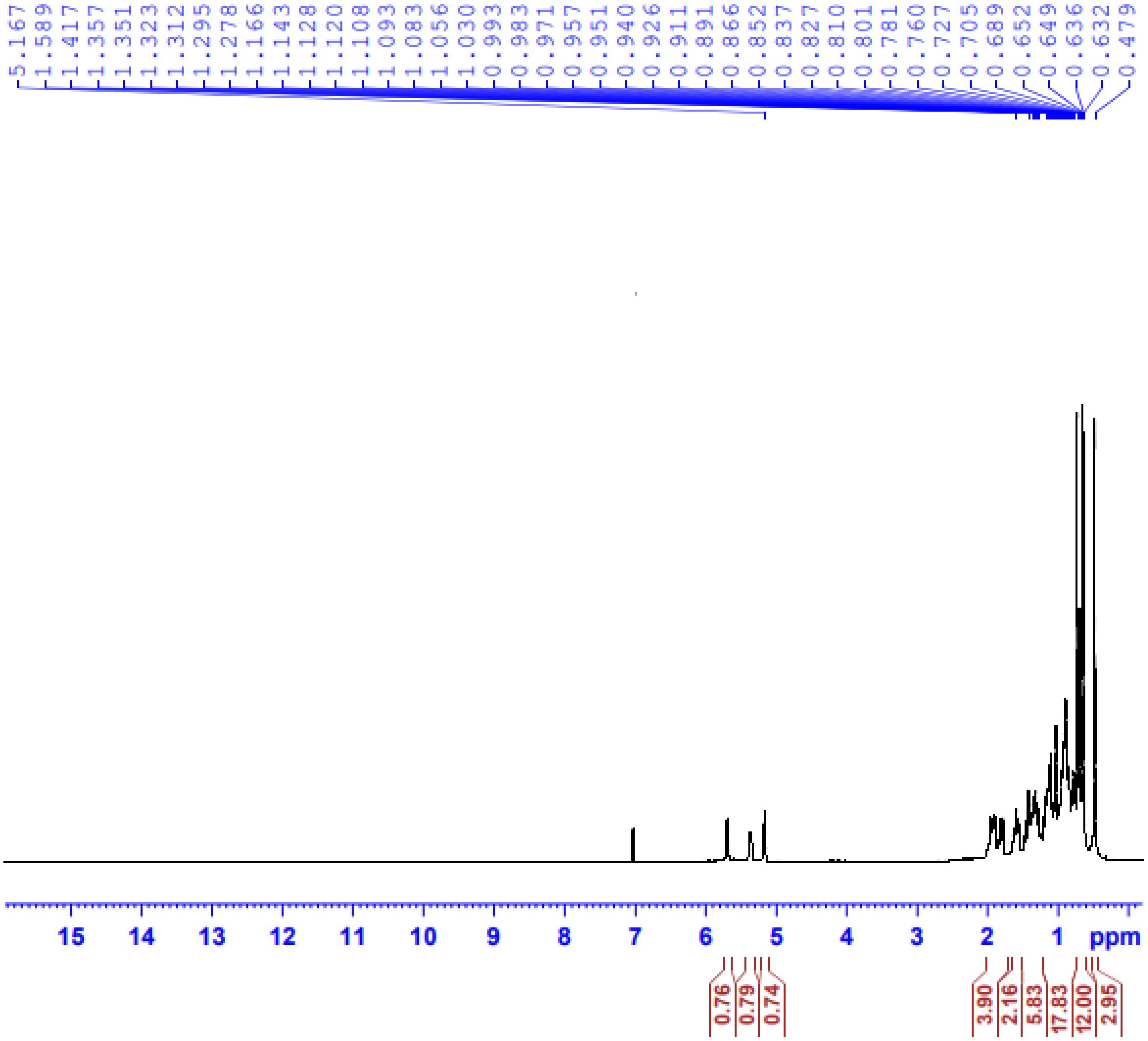

Fig. S2. ^{1}H NMR spectrum (400 MHz, $CDCl_3$) of compound **9**. A spectrum for this compound was previously published in [9], but incorrectly assigned to 3α-bromocholest-5-ene.

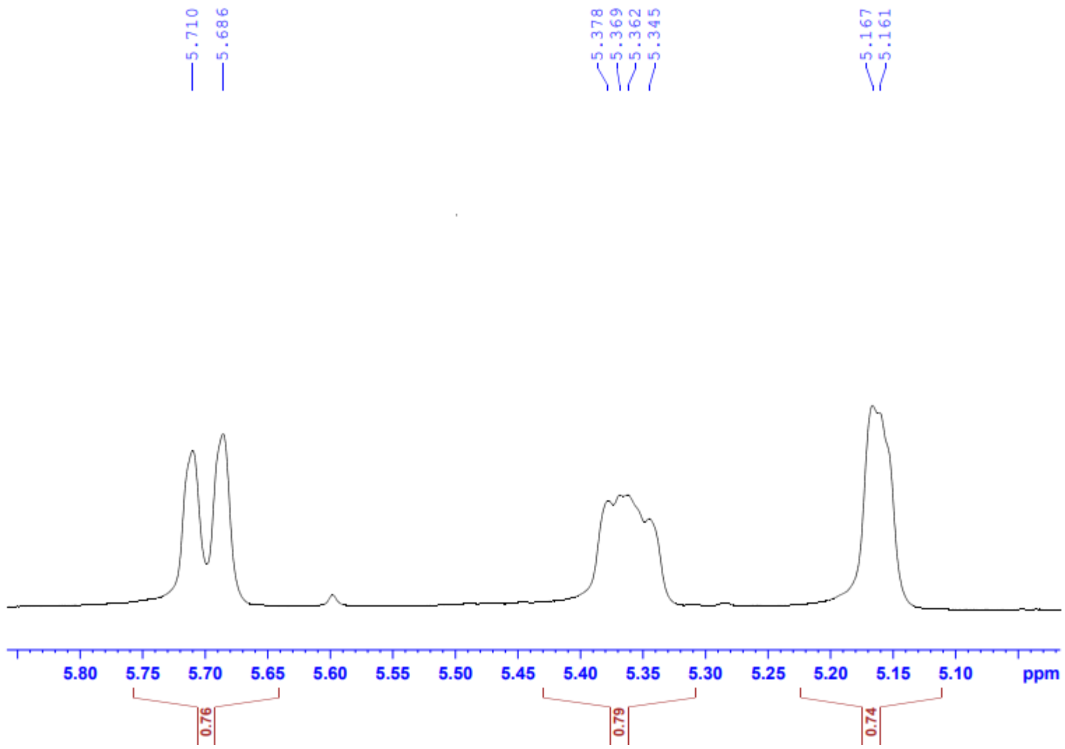

Fig. S3. Cross-section in the ^{1}H NMR spectrum (400 MHz, $CDCl_3$) of compound **9**.

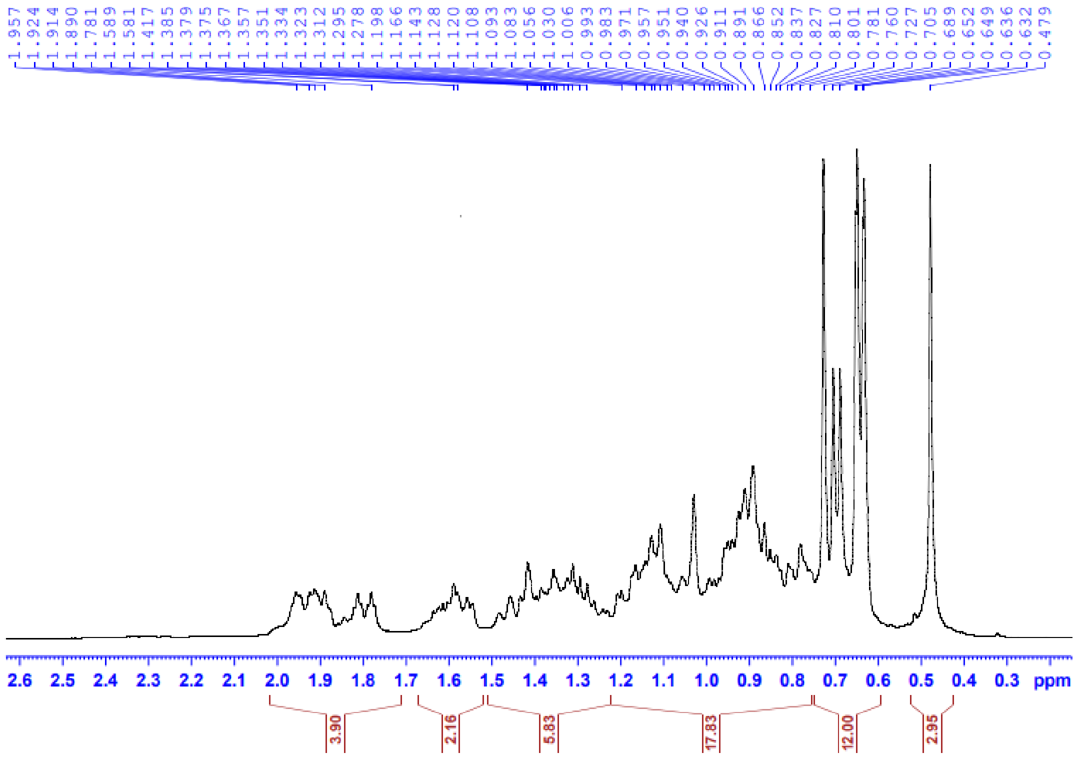

Fig. S4. Cross-section in the ^{1}H NMR spectrum (400 MHz, $CDCl_3$) of compound **9**.

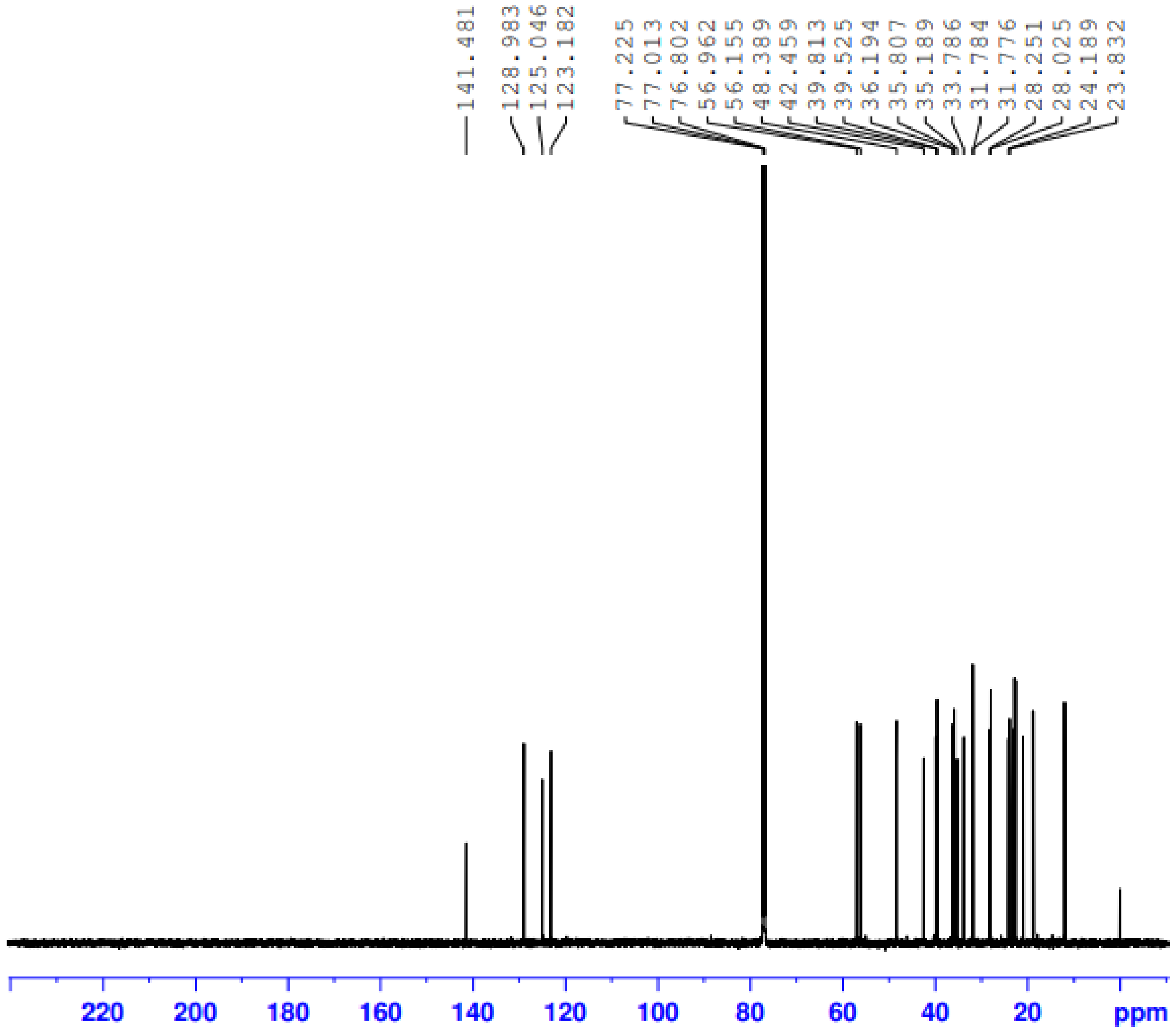

Fig. S5. ^{13}C {^1H} NMR spectrum (150 MHz, $CDCl_3$) of compound **9**. This spectrum was slightly adapted from [9] ("Synthesis, antimicrobial and cytotoxicity evaluation of new cholesterol congeners", © 2015 M. R. E. S. Aly et al., published by the Beilstein-Institut, distributed under the terms of the Creative Commons Attribution 2.0 Generic License, https://creativecommons.org/licenses/by/2.0). This spectrum was incorrectly assigned to 3α-bromocholest-5-ene in [9].

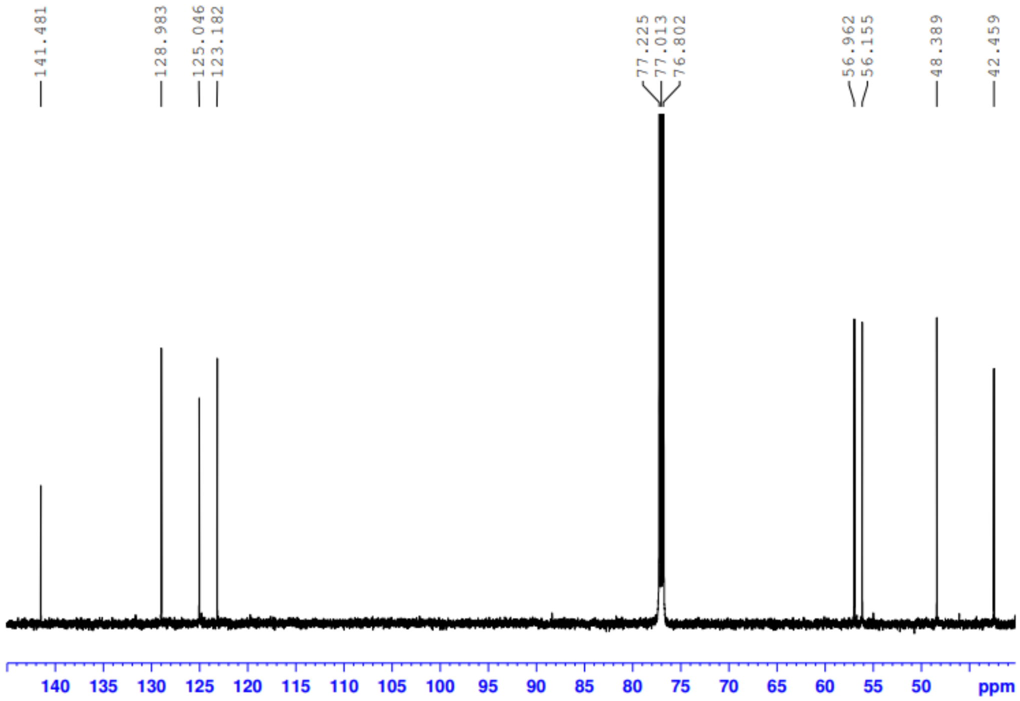

Fig. S6. Cross-section in the ^{13}C {^{1}H} NMR spectrum of compound **9**.

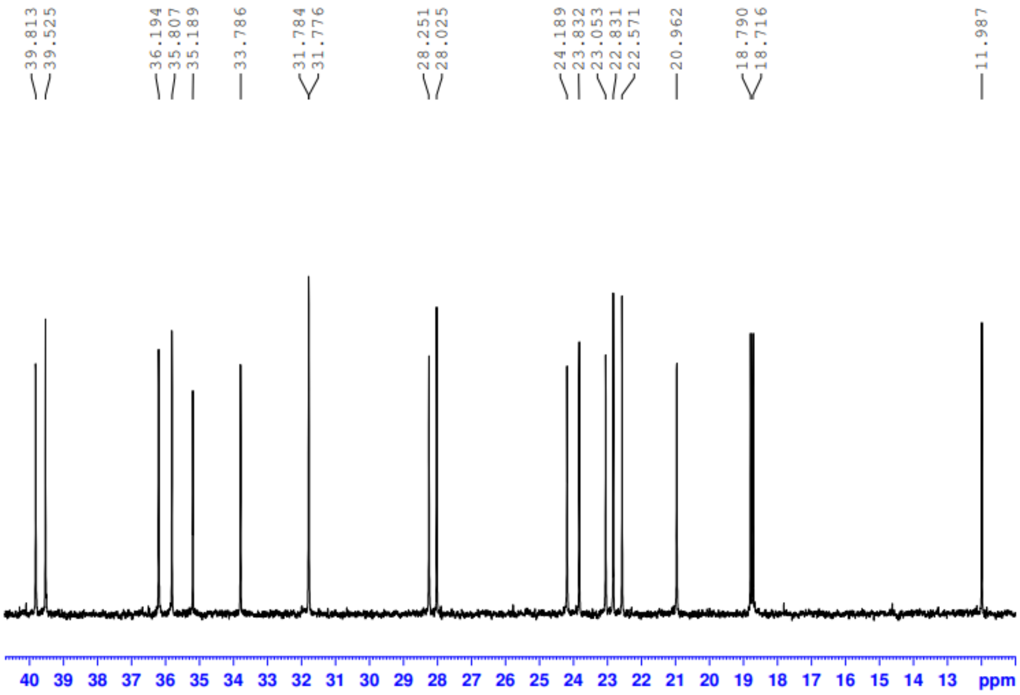

Fig. S7. Cross-section in the ^{13}C {^{1}H} NMR spectrum of compound **9**.

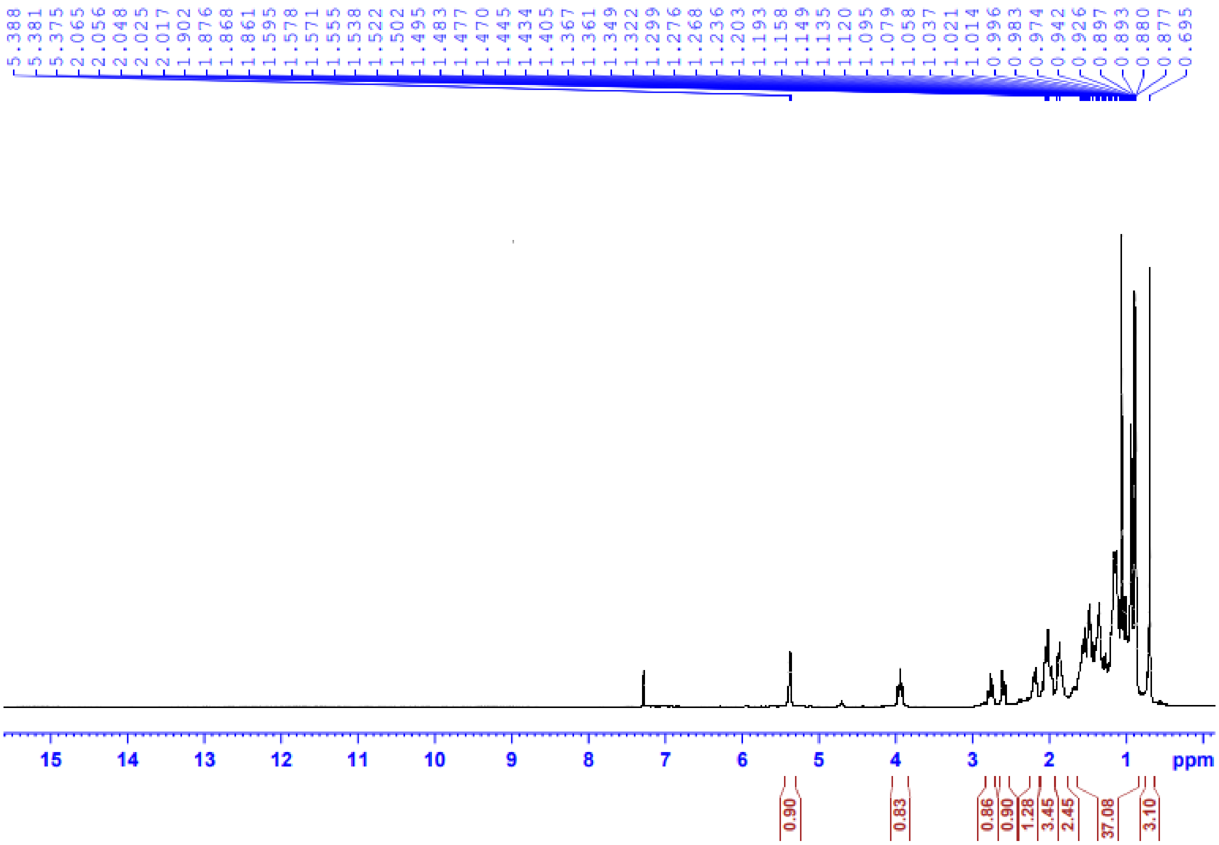

Fig. S8. ^{1}H NMR spectrum (400 MHz, $CDCl_3$) of compound **4**.

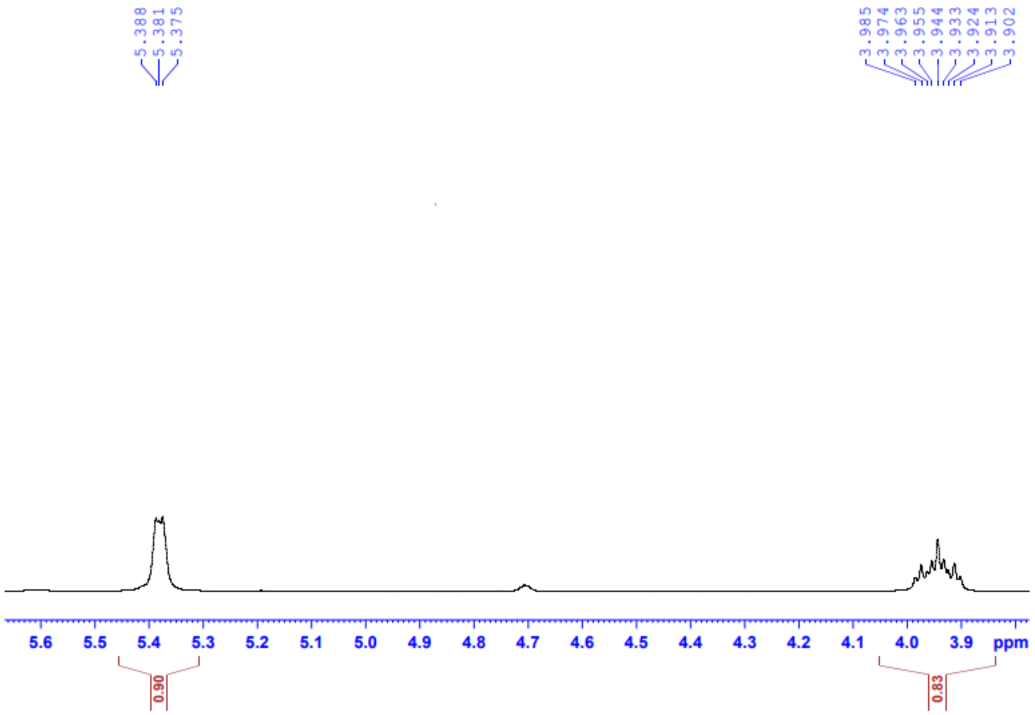

Fig. S9. Cross-section in the ^{1}H NMR spectrum of compound **4**.

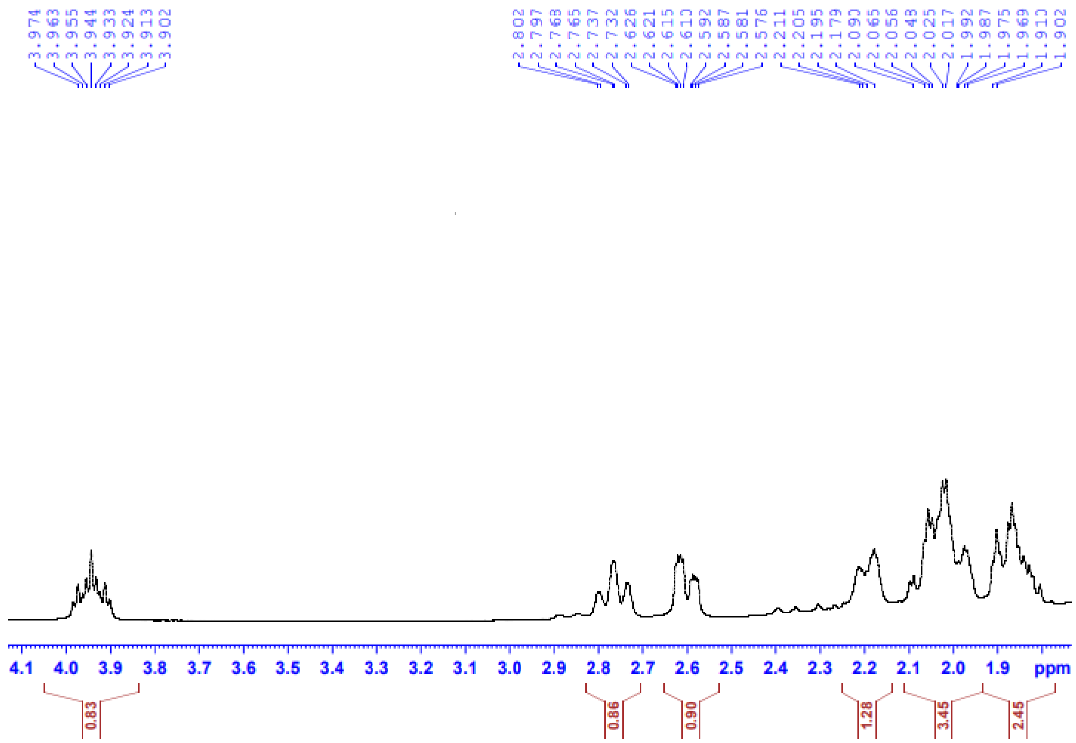

Fig. S10. Cross-sections in the ^{1}H NMR spectrum of compound **4**.

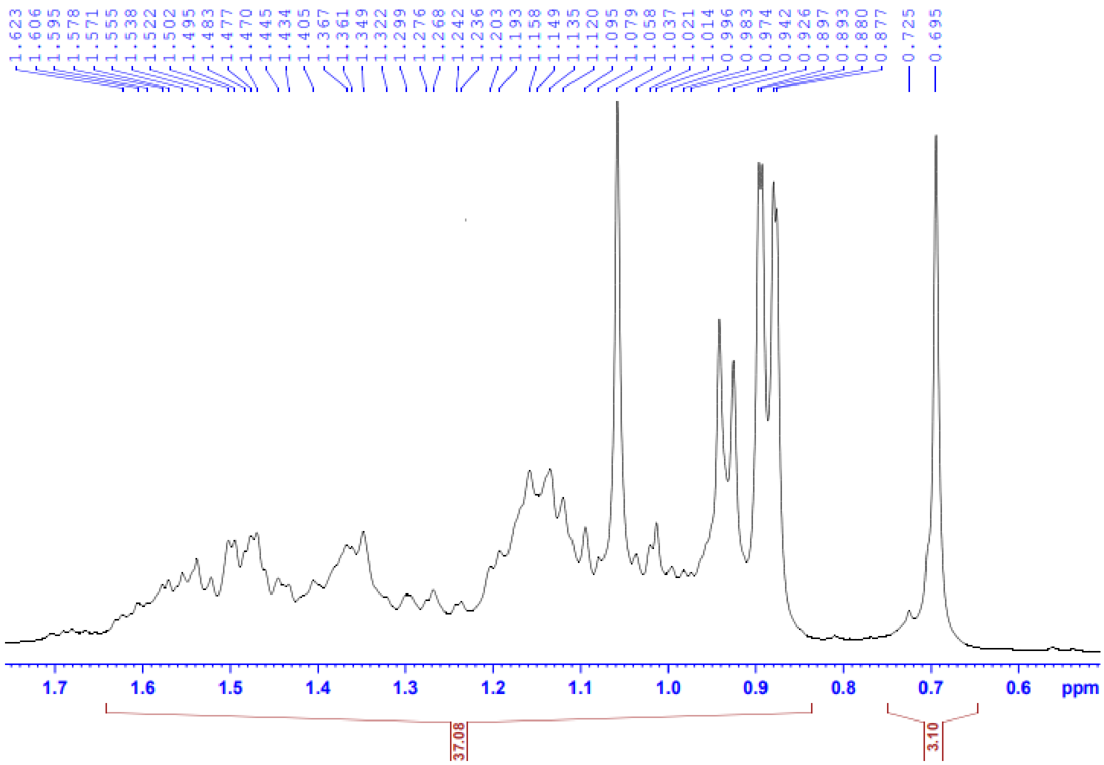

Fig. S11. Cross-sections in the ^{1}H NMR spectrum of compound **4**.

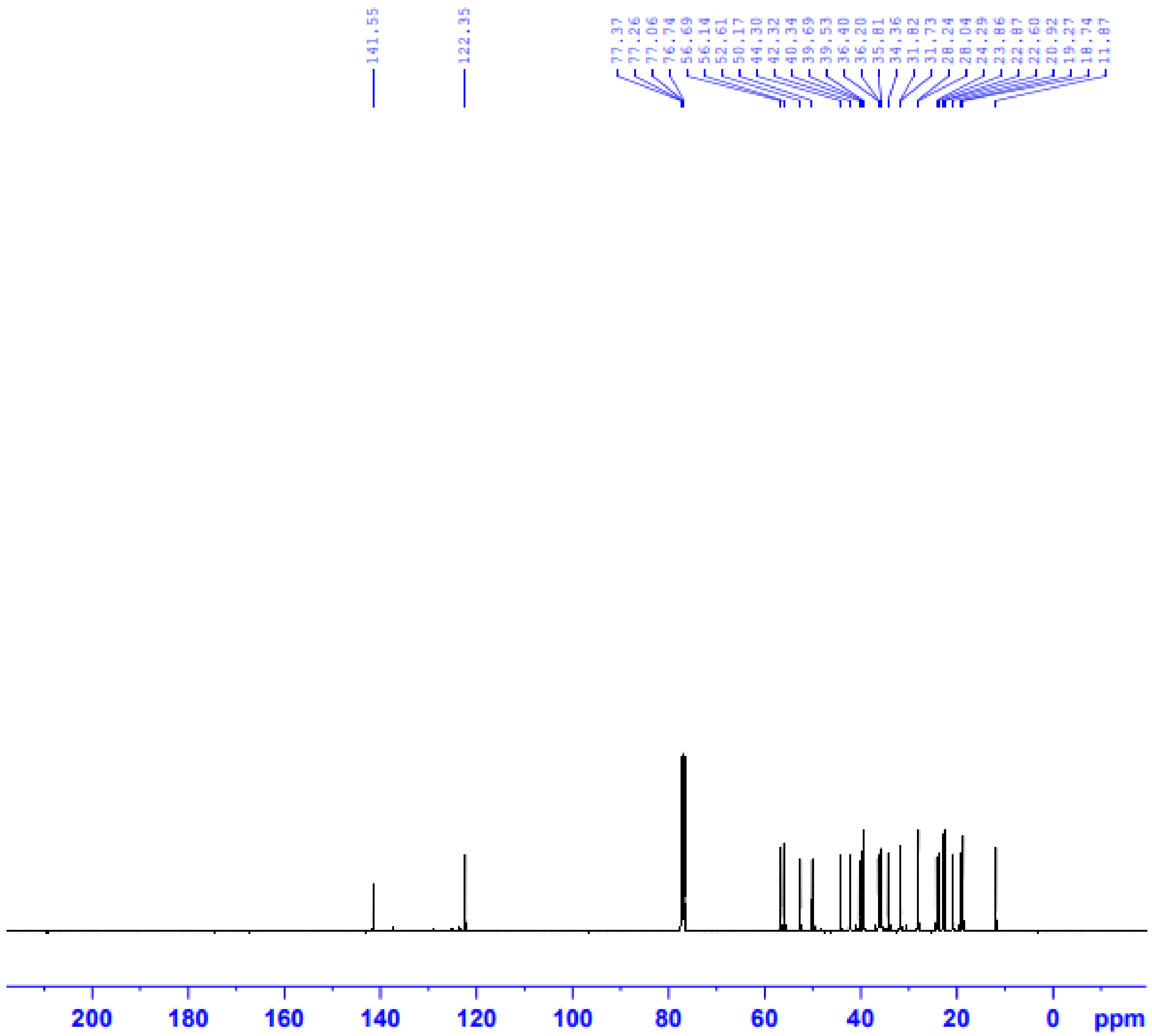

Fig. S12. ^{13}C {^{1}H} NMR spectrum (100 MHz, $CDCl_3$) of compound **4**.

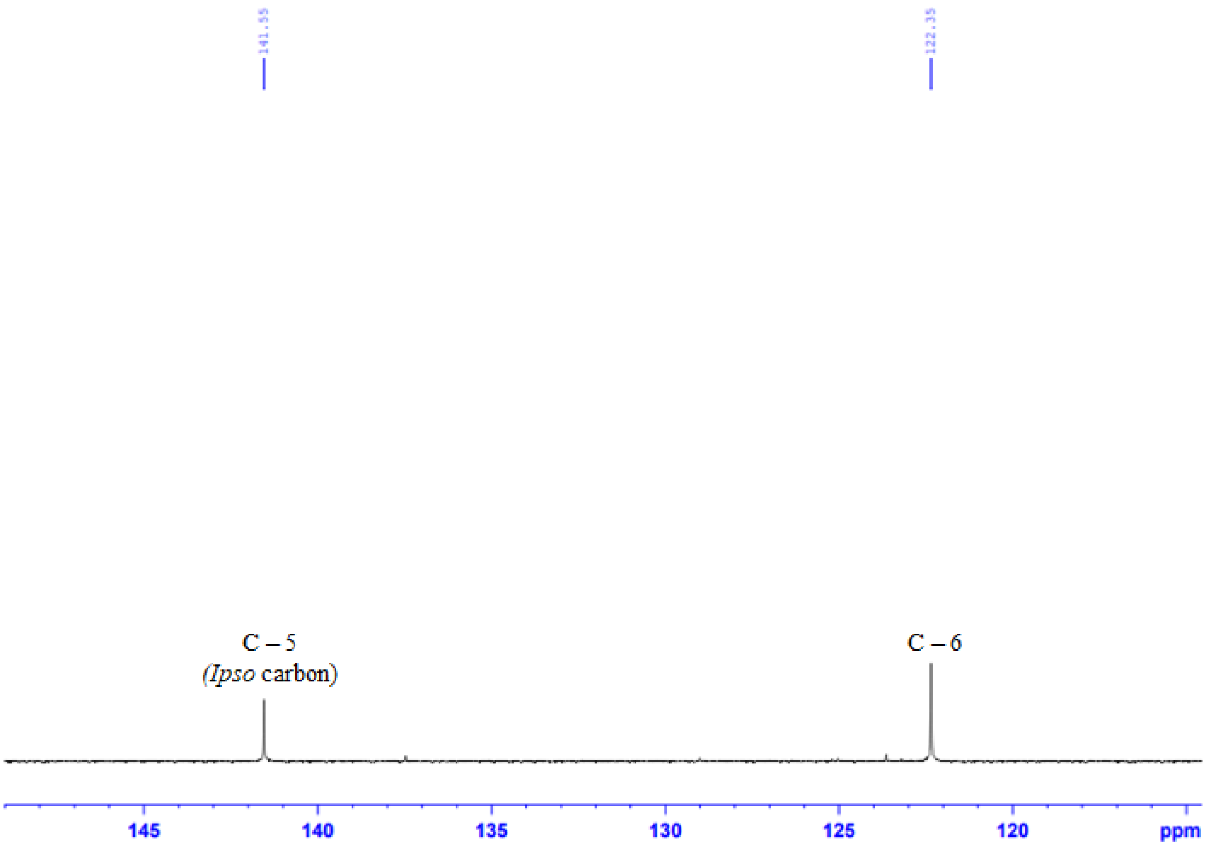

Fig. S13. Cross-section in the ^{13}C {^{1}H} NMR spectrum (100 MHz, $CDCl_3$) of compound **4**.

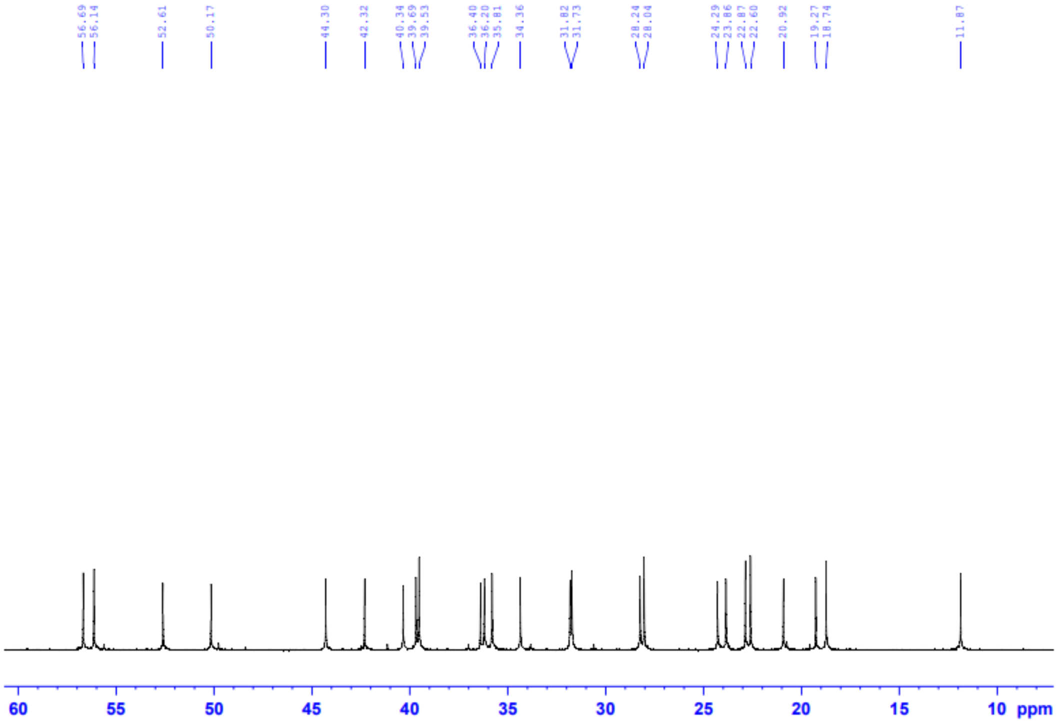

Fig. S14. Cross-section in the ^{13}C {^{1}H} NMR spectrum (100 MHz, $CDCl_3$) of compound **4**.

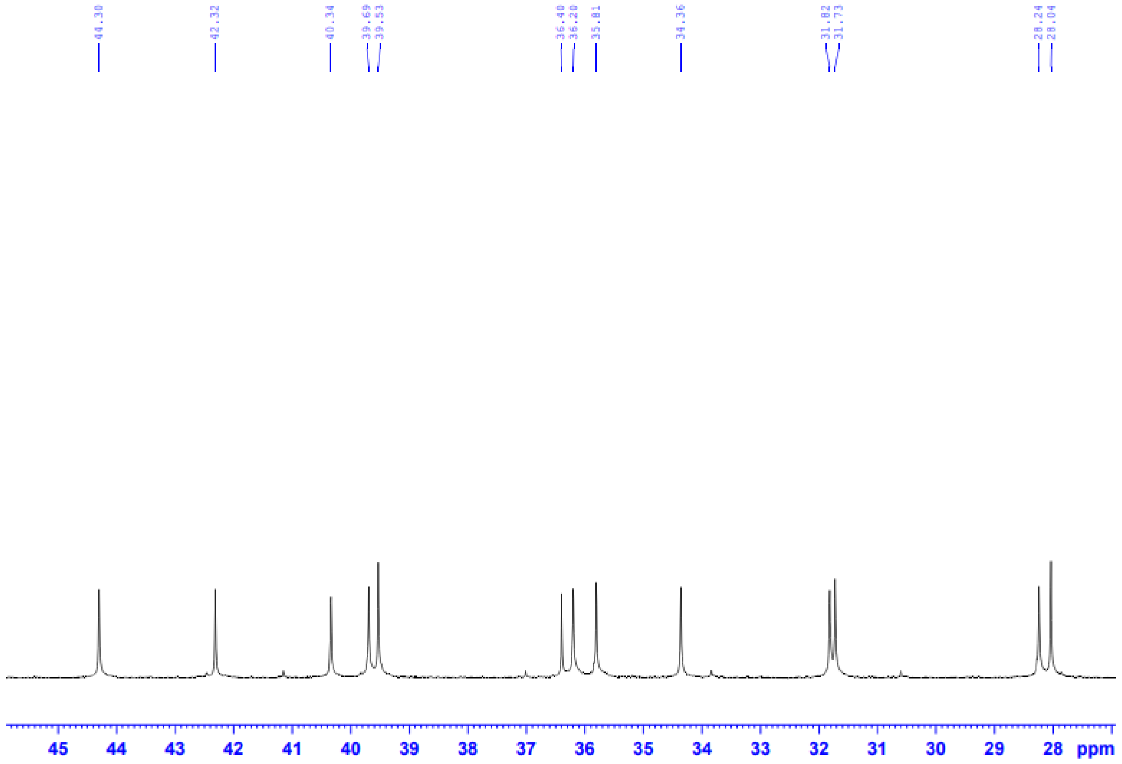

Fig. S15. Cross-section in the ^{13}C {^{1}H} NMR spectrum (100 MHz, $CDCl_3$) of compound **4**.

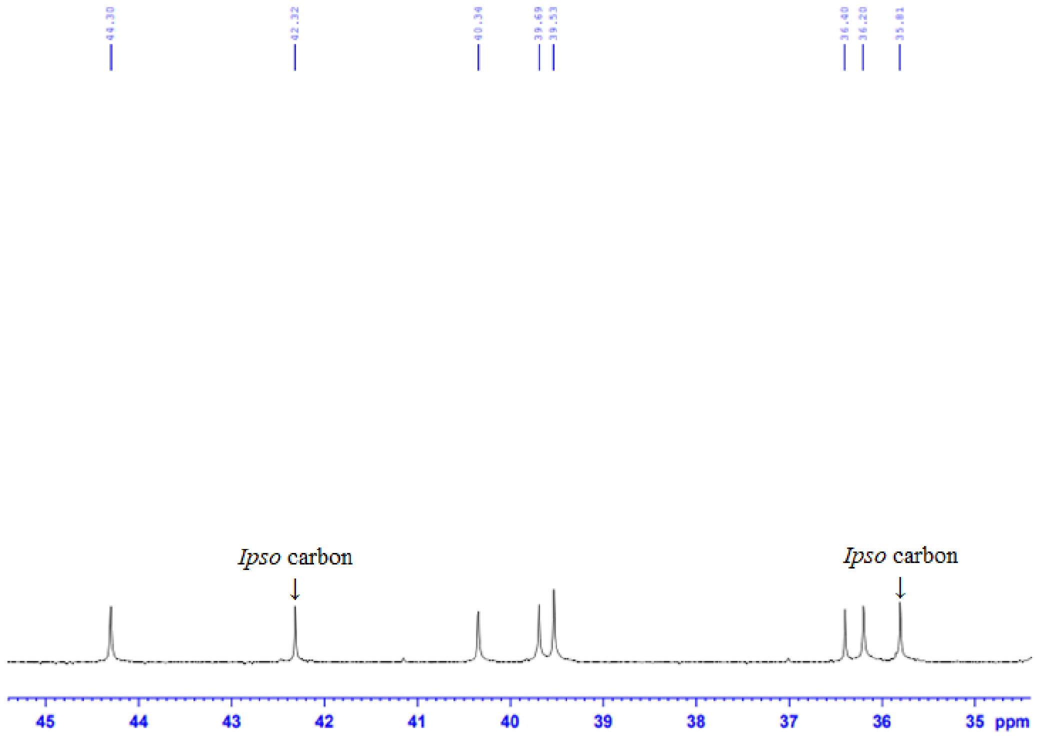

Fig. S16. Cross-section in the ^{13}C {^{1}H} NMR spectrum (100 MHz, $CDCl_3$) of compound **4**.

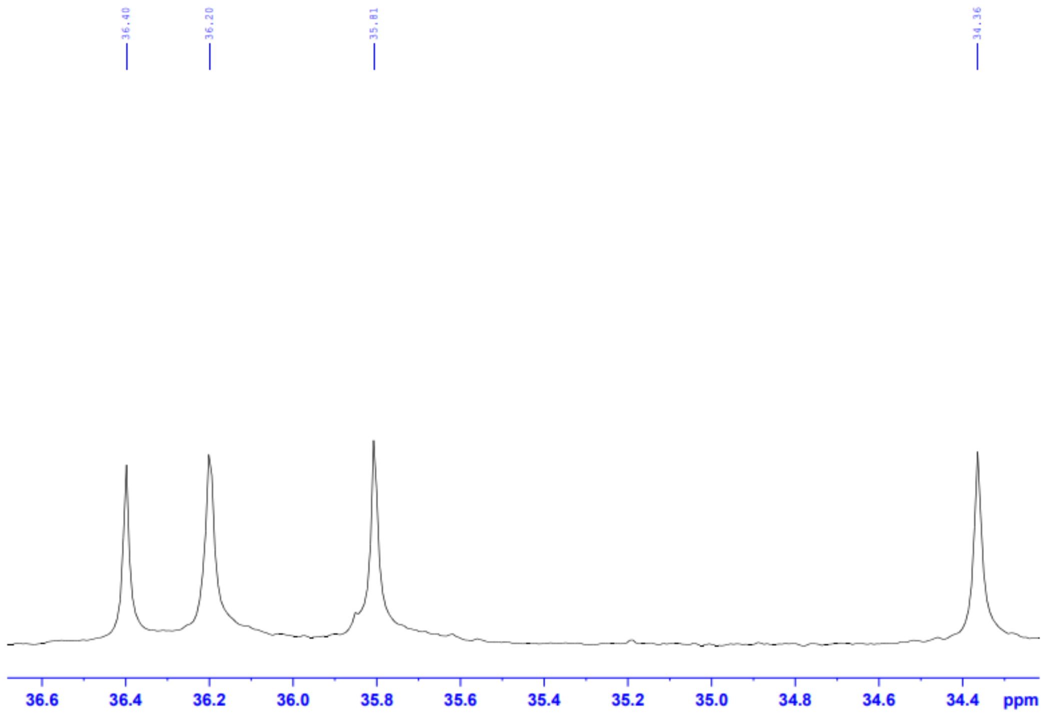

Fig. S17. Cross-section in the ^{13}C {^{1}H} NMR spectrum (100 MHz, $CDCl_3$) of compound **4**.

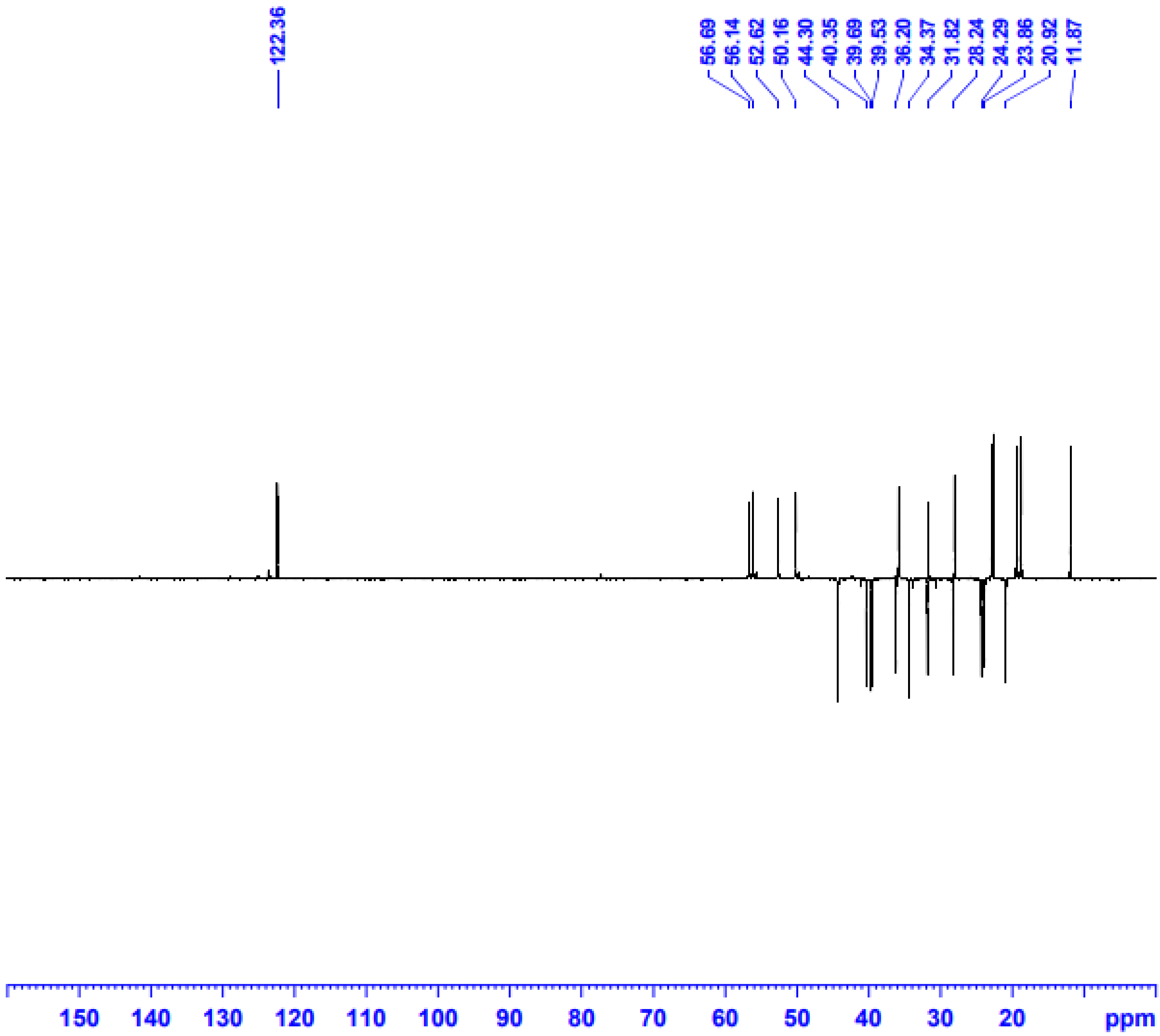

Fig. S18. DEPT-135° spectrum (100 MHz, $CDCl_3$) of compound **4**.

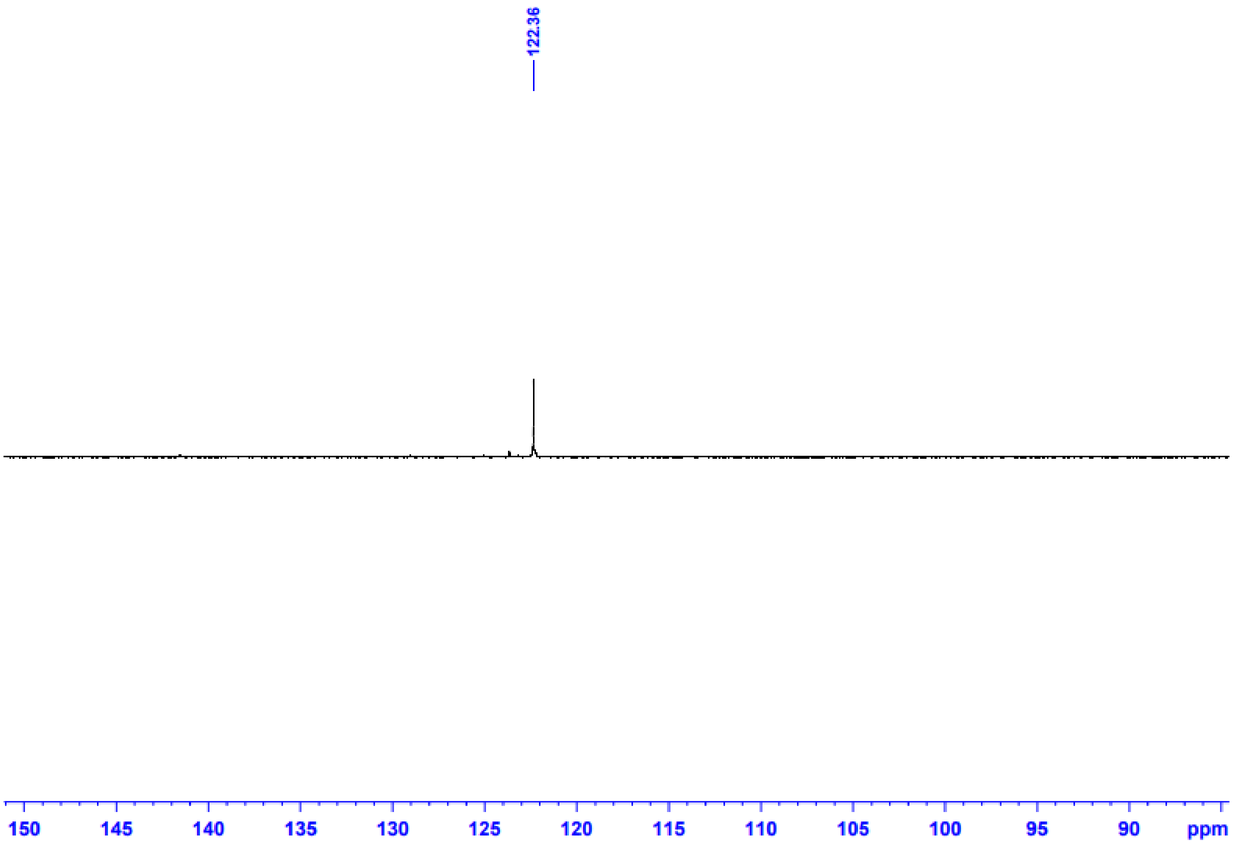

Fig. S19. Cross-section in the DEPT-135° spectrum (100 MHz, $CDCl_3$) of compound **4**.

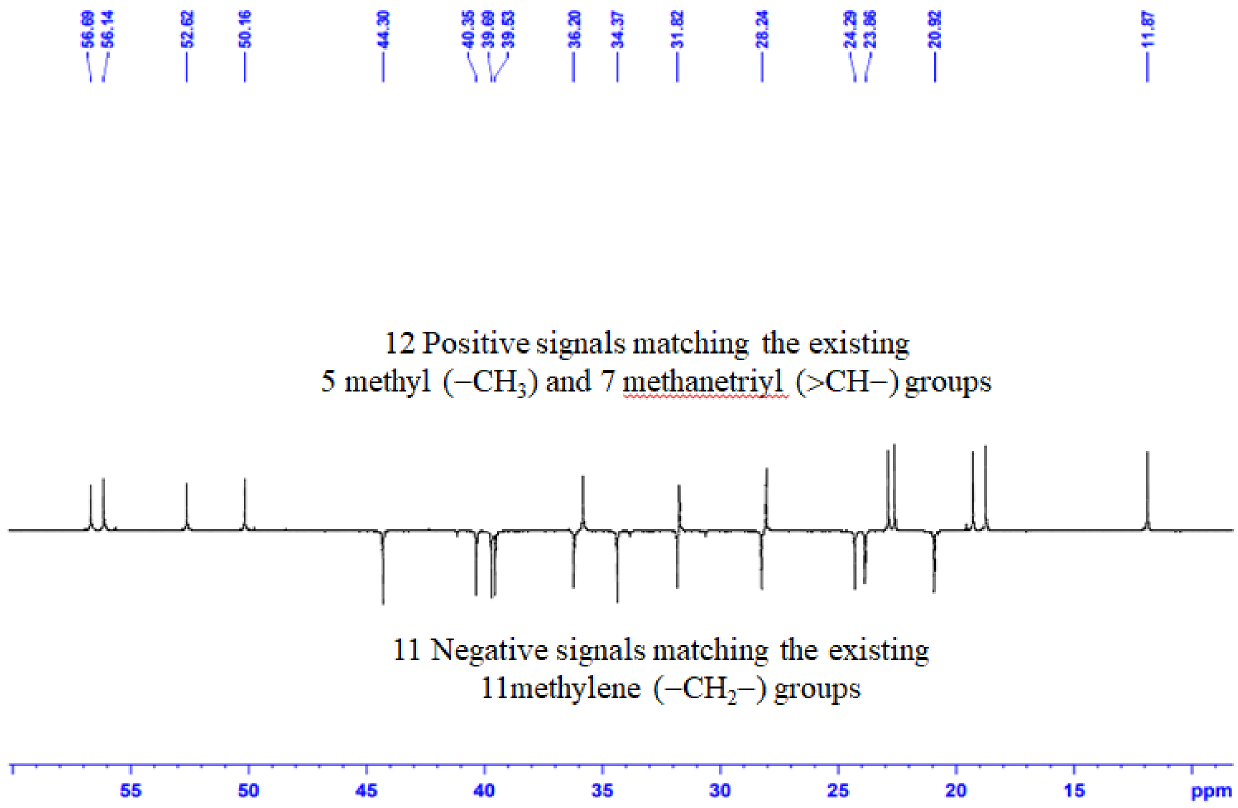

Fig. S20. Cross-section in the DEPT-135° spectrum (100 MHz, $CDCl_3$) of compound **4**.

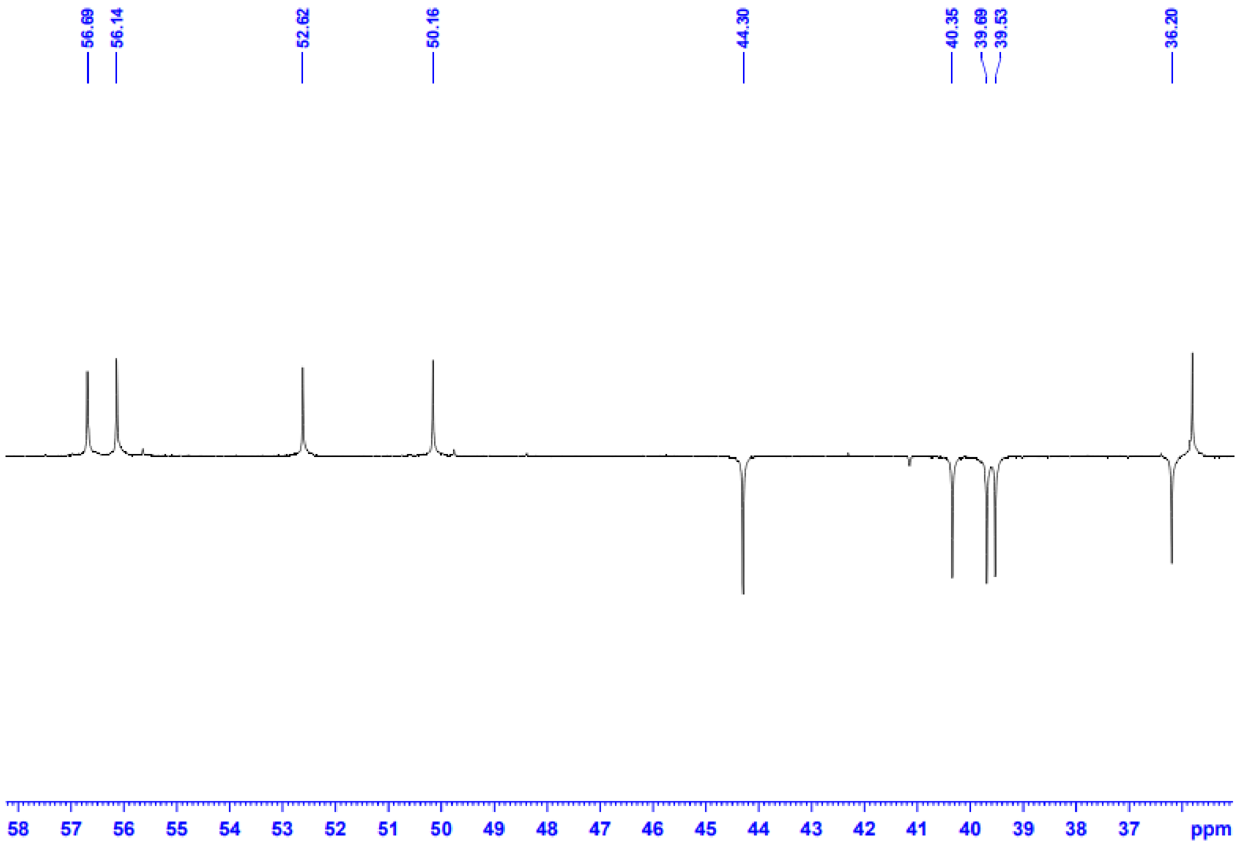

Fig. S21. Cross-section in the DEPT-135° spectrum (100 MHz, $CDCl_3$) of compound **4**.

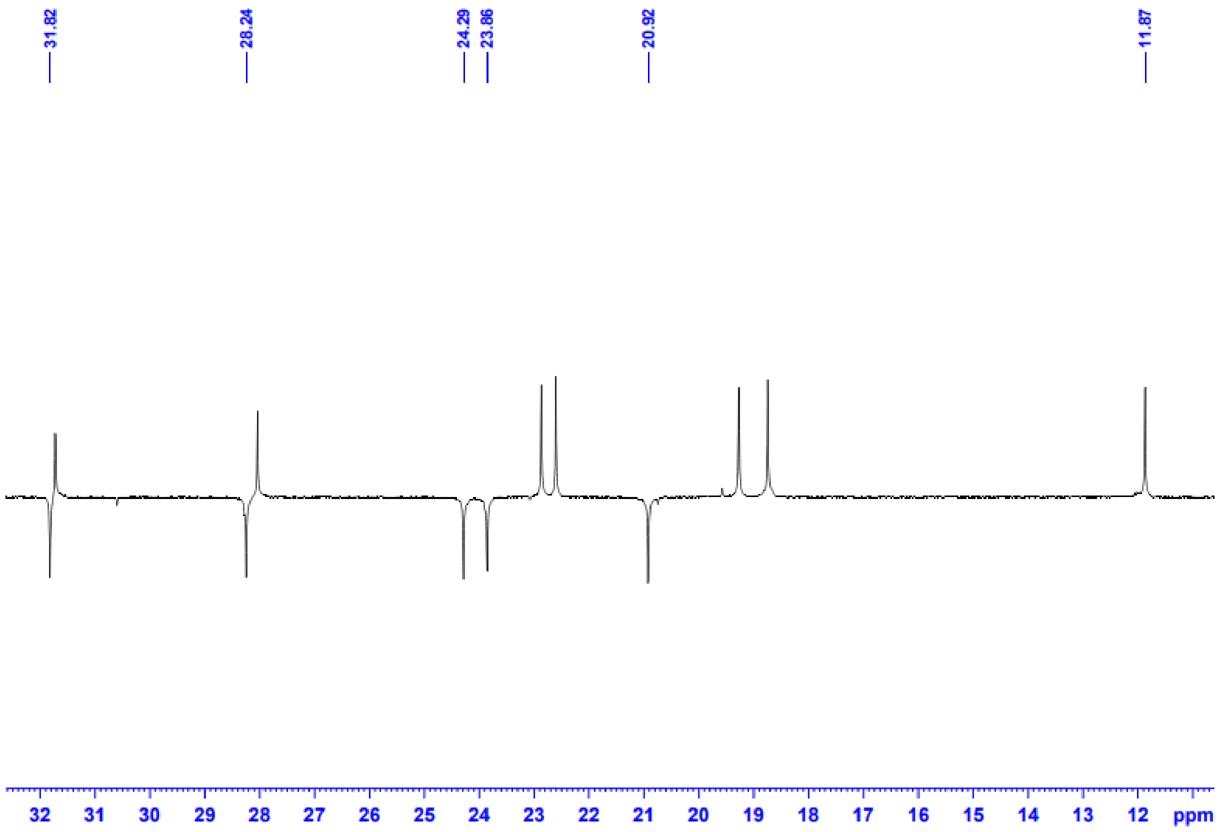

Fig. S22. Cross-section in the DEPT-135º spectrum (100 MHz, $CDCl_3$) of compound **4**.

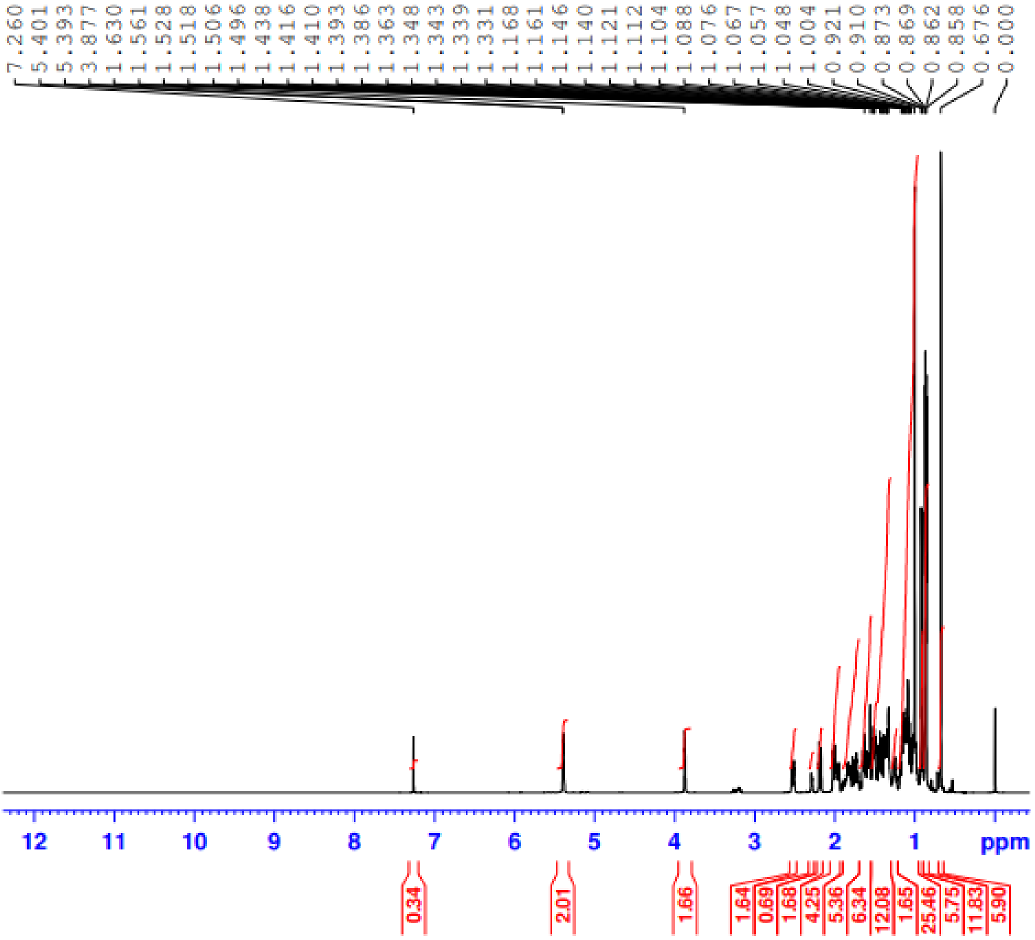

Fig. S23. ^{1}H NMR spectrum (600 MHz, $CDCl_3$) of compound **5**. This spectrum was slightly adapted from [9] (“Synthesis, antimicrobial and cytotoxicity evaluation of new cholesterol congeners”, © 2015 M. R. E. S. Aly et al., published by the Beilstein-Institut, distributed under the terms of the Creative Commons Attribution 2.0 Generic License, https://creativecommons.org/licenses/by/2.0). This spectrum was incorrectly assigned to 3β-azidocholest-5-ene in [9]. The weak signal at δ = 3.20 (H-3_{β}) belongs to the β-epimer (ca. 15%) [10].

.

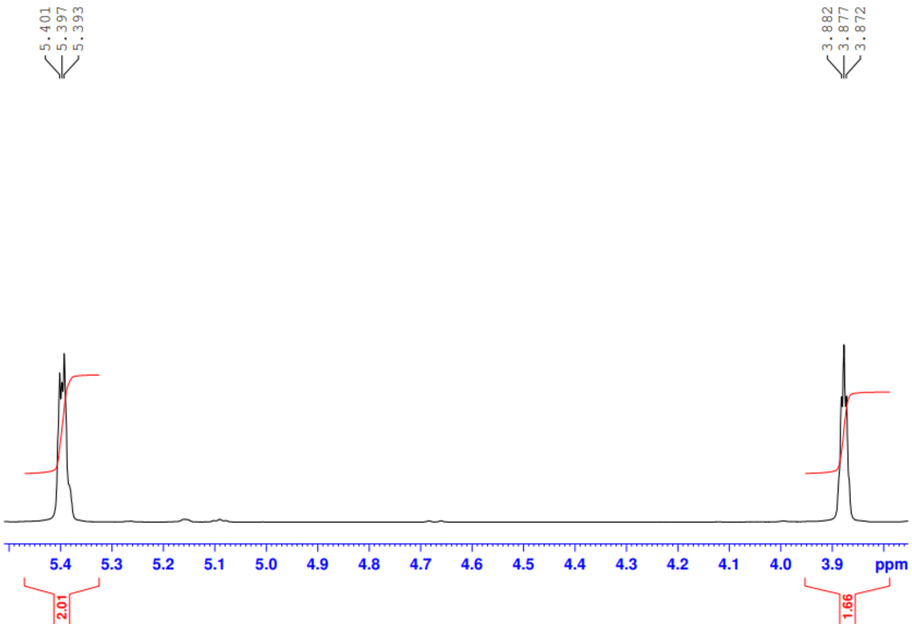

Fig. S24. Cross-section in the ^{1}H NMR spectrum of compound **5**.

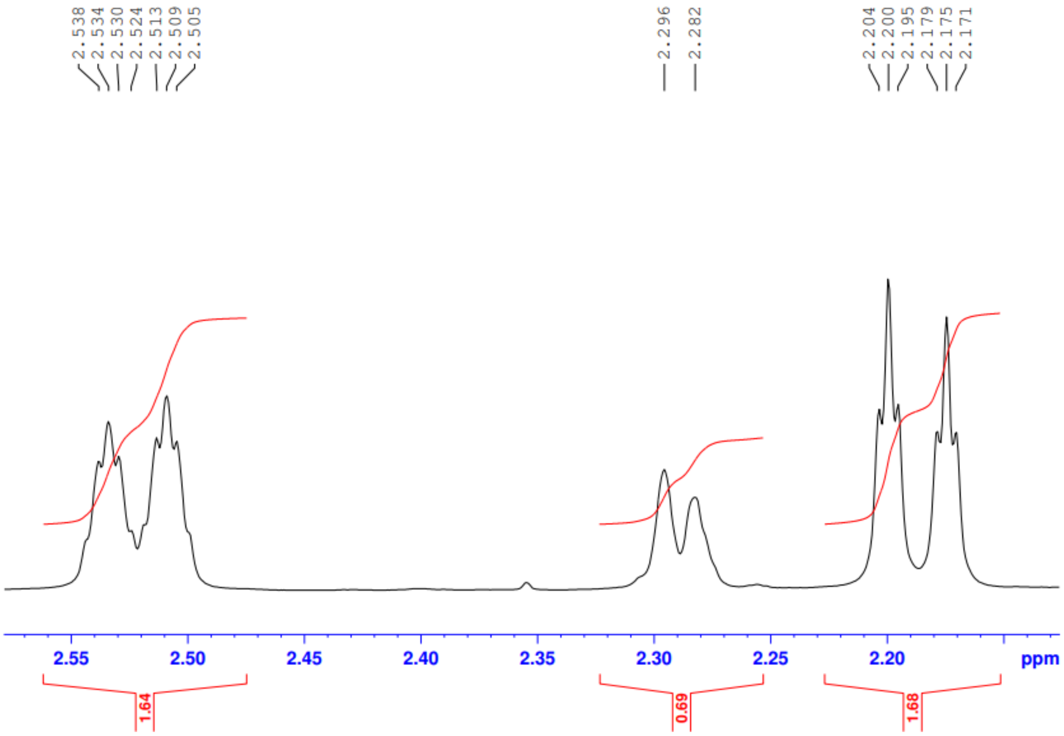

Fig. S25. Cross-section in the ^{1}H NMR spectrum of compound **5**. The signal at δ = 2.28 ppm arise from two protons of the β-epimer (ca. 15%) [10].

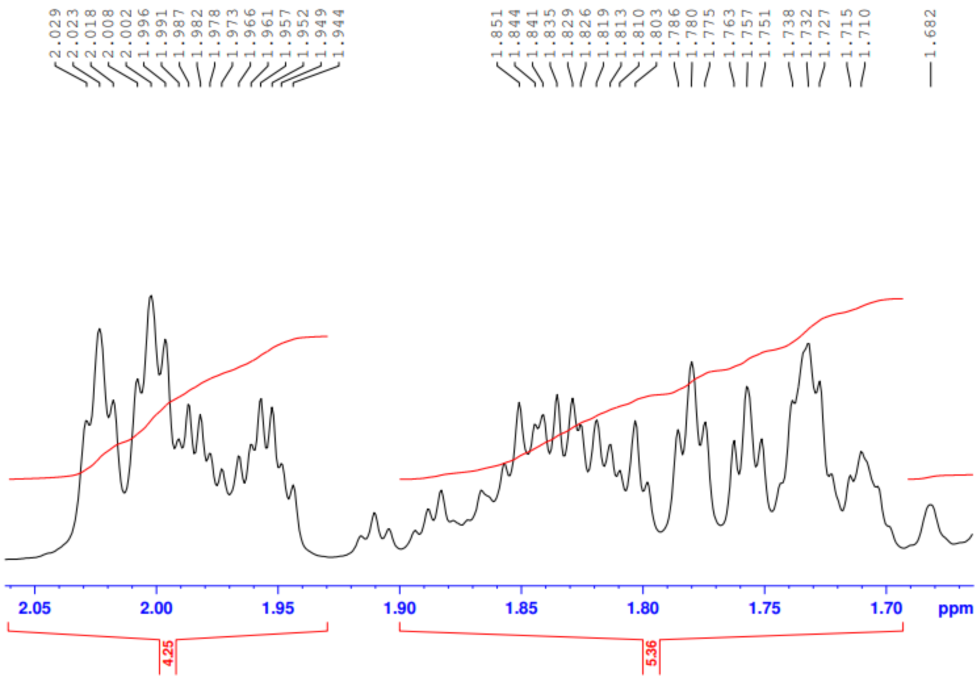

Fig. S26. Cross-section in the ^{1}H NMR spectrum of compound **5**.

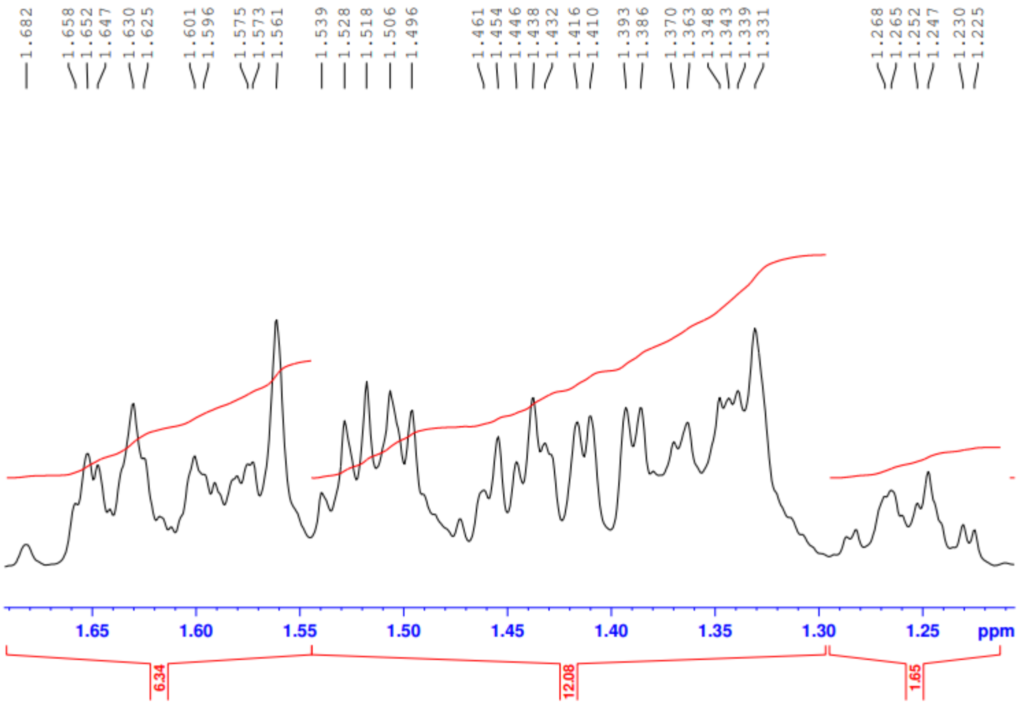

Fig. S27. Cross-section in the ^{1}H NMR spectrum of compound **5**.

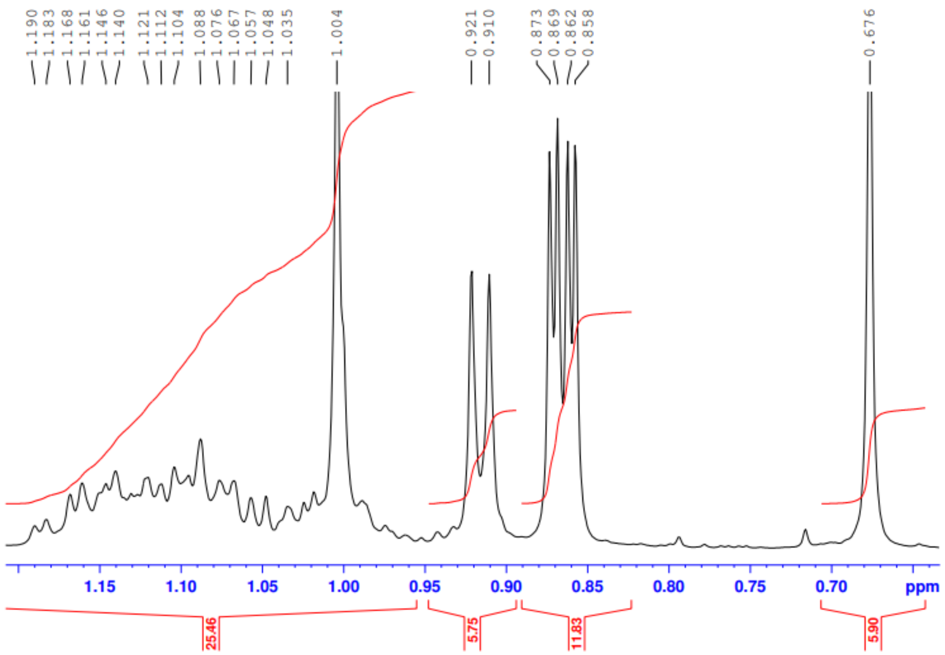

Fig. S28. Cross-section in the ^{1}H NMR spectrum of compound **5**.

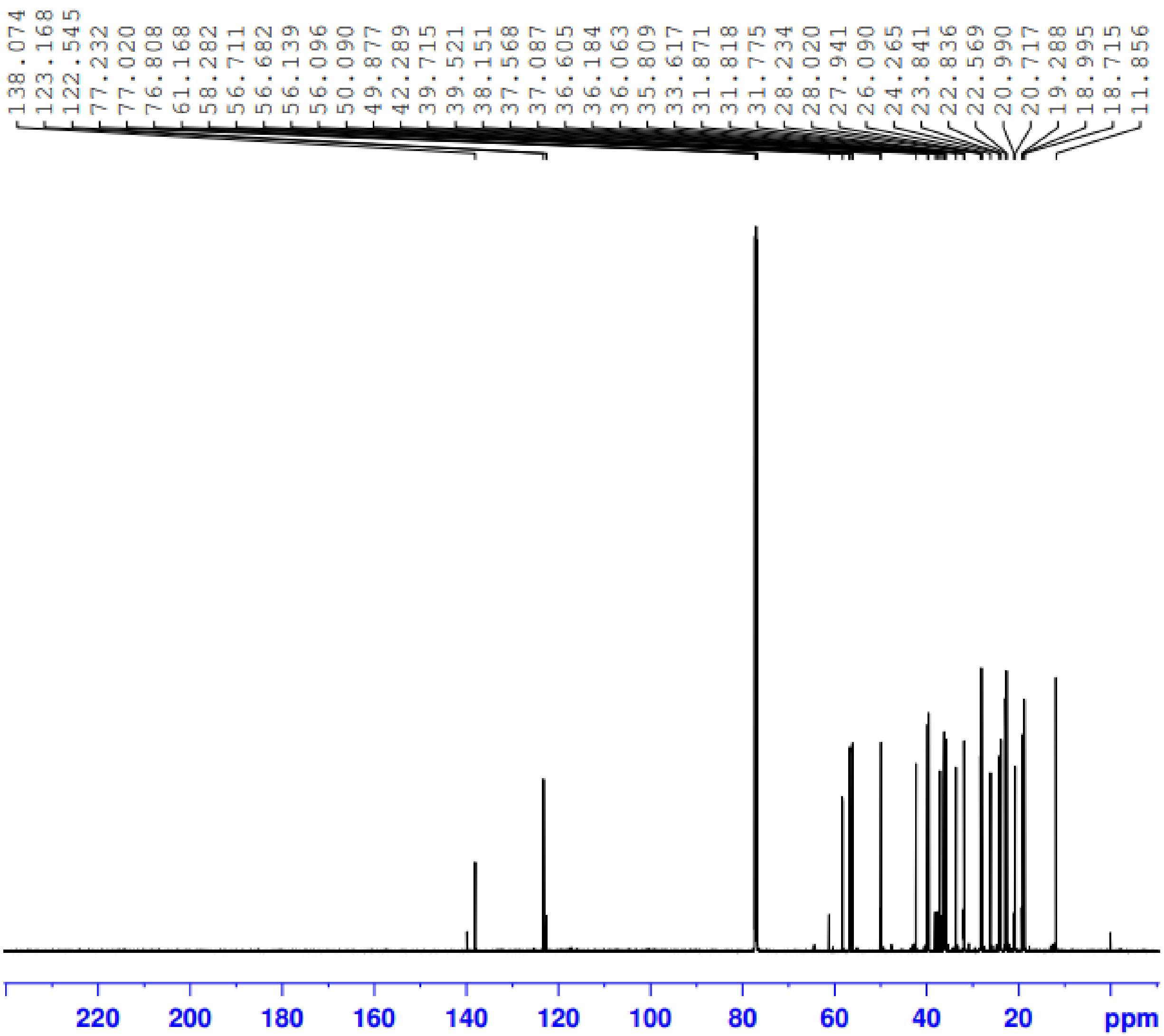

Fig. S29. ^{13}C {^{1}H} NMR spectrum (150 MHz, $CDCl_3$) of compound **5**. This spectrum was slightly adapted from [9] ("Synthesis, antimicrobial and cytotoxicity evaluation of new cholesterol congeners", © 2015 M. R. E. S. Aly et al., published by the Beilstein-Institut, distributed under the terms of the Creative Commons Attribution 2.0 Generic License, https://creativecommons.org/licenses/by/2.0). This spectrum was incorrectly assigned to 3β-azidocholest-5-ene in [9].

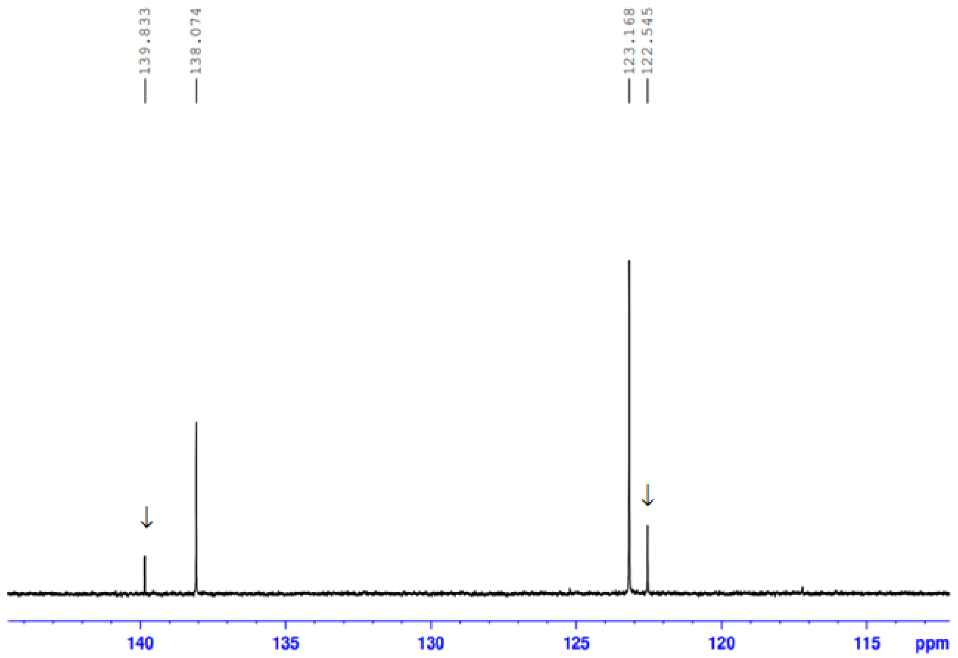

Fig. S30. Cross-section in the ^{13}C {^{1}H} NMR spectrum (150 MHz, $CDCl_3$) of compound **5**. The arrows here and in the next cross-sections denote to the signals arising from the presence of trace of 3β-azidocholest-5-ene [10].

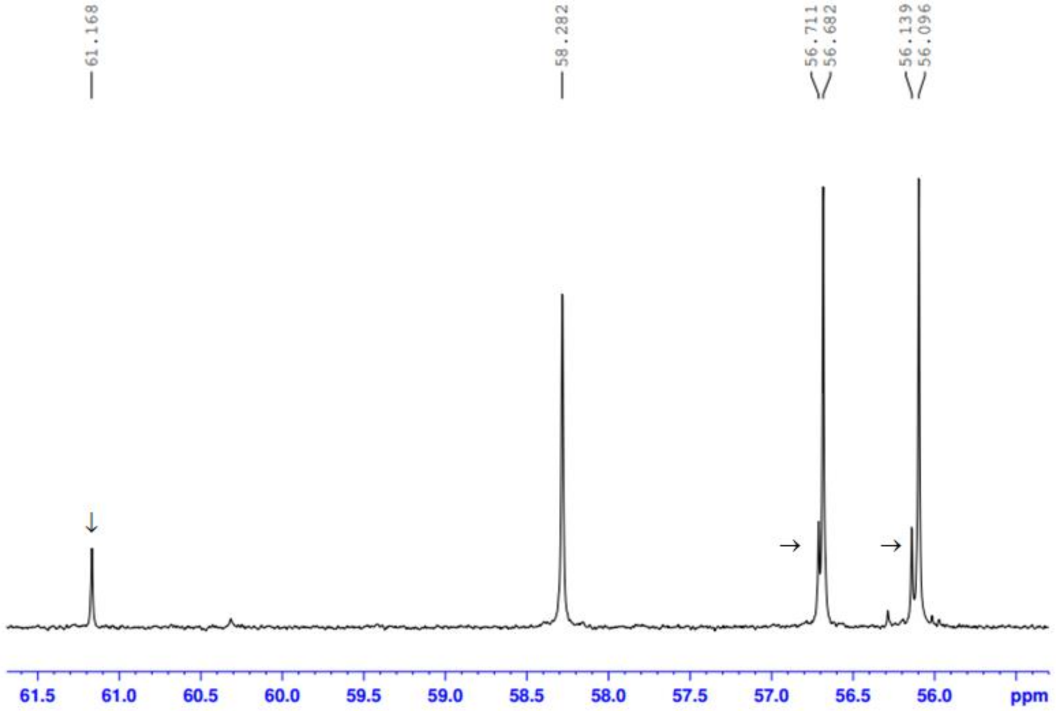

Fig. S31. Cross-section in the ^{13}C {^{1}H} NMR spectrum (150 MHz, $CDCl_3$) of compound **5**.

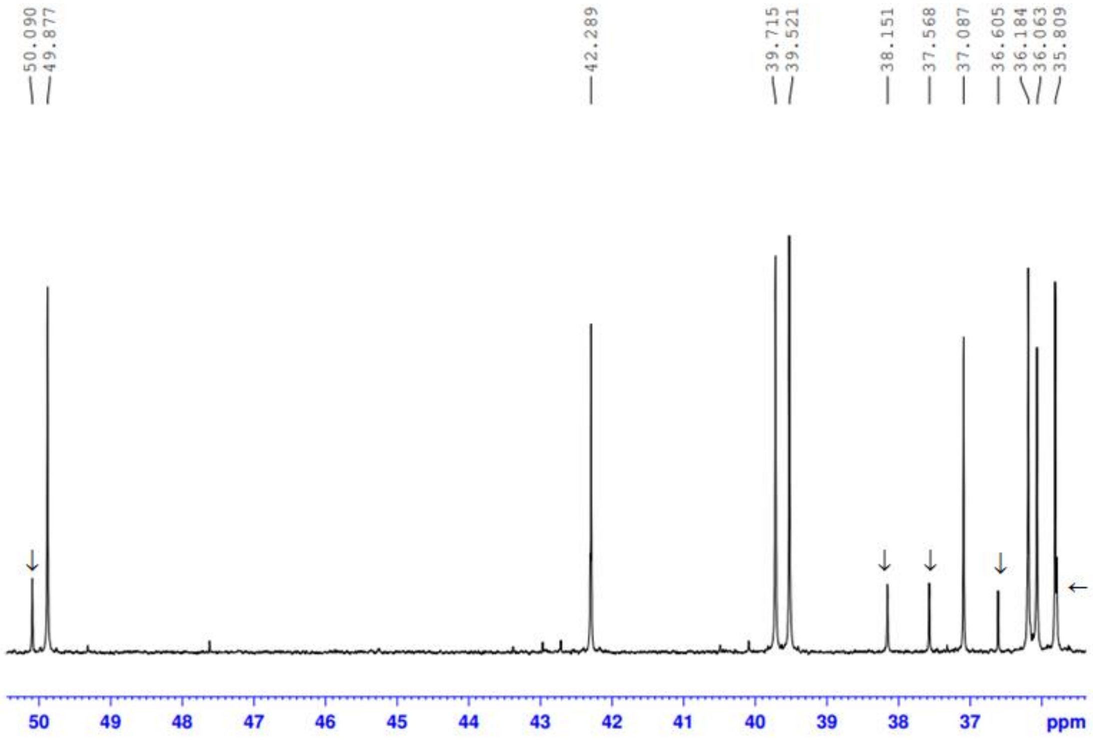

Fig. S32. Cross-section in the ^{13}C {^{1}H} NMR spectrum (150 MHz, $CDCl_3$) of compound **5**.

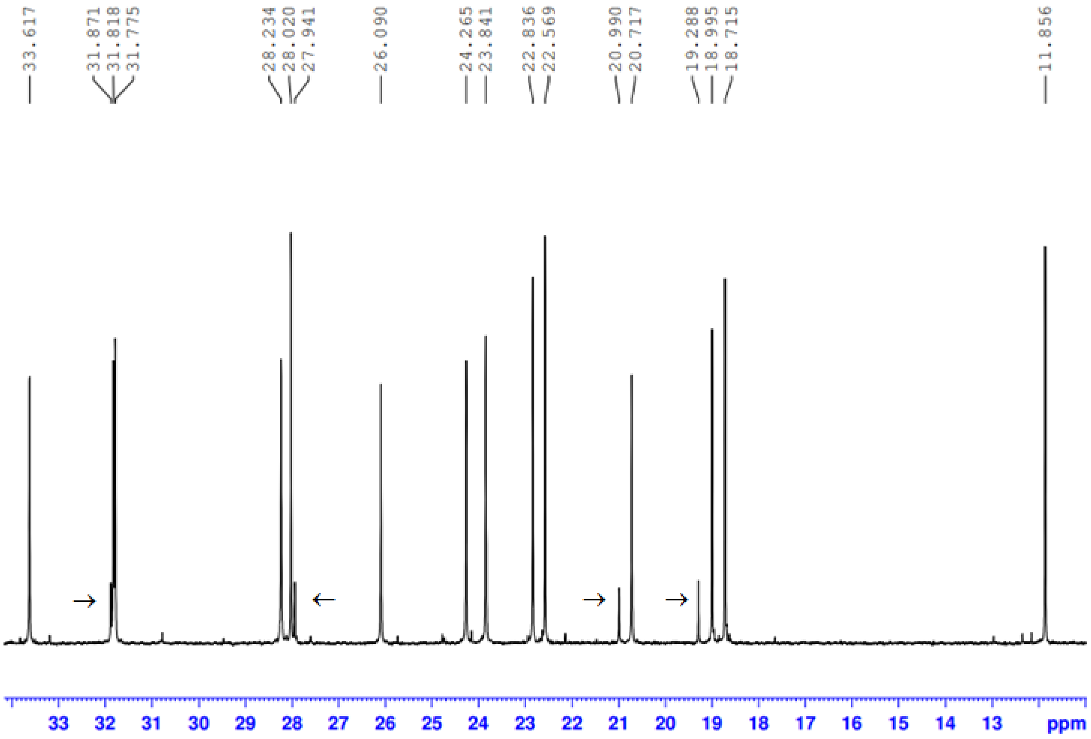

Fig. S33 . Cross-section in the ^{13}C {^{1}H} NMR spectrum (150 MHz, $CDCl_3$) of compound **5**.

III. References

1. Hooft, R.W.W. *COLLECT* **1998**, Nonius BV, Delft, The Netherlands.
2. Otwinowski, Z.; Minor, W. *Methods in Enzymology, Macromolecular Crystallography, Part A* **1997**, *276*, 307–326. Edited by Carter Jr C. W.; Sweet, R. M. New York: Academic Press.
3. Sheldrick, G. M. *SADABS* Version 2008/2 **1996**, University of Göttingen, Germany.
4. Rigaku Oxford Diffraction, **2018**, *CrysAlisPro* Software system, Rigaku Corporation, Oxford, UK.
5. Sheldrick, G. M. *Acta Crystallogr. Sect. A* **2015**, *71*, 3–8.
6. Dolomanov, O. V; Bourhis, L. J.; Gildea, R. J.; Howard, J. A. K.; Puschmann, H. *J. Appl. Crystallogr.* **2009**, *42*, 339–341.
7. Sheldrick, G. M. *Acta Crystallogr. Sect. C* **2015**, *71*, 3–8.
8. Houston, T. A.; Quader, S.; Boyd, S. E.; Jenkins, I. D.; Healy, P. C. *Acta Cryst.* **2008**, *E64*, o1738.
9. Aly, M. R. E.; Saad, H. A.; Abdel Hafez, S. H. *Beilstein J. Org. Chem.* **2015**, *11*, 1922–1932. doi.org/10.3762/bjoc.11.208
10. Sun, Q.; Cai, S.; Peterson, B. R. *Org. Lett.* **2009**, *11*, 567–570. doi.org/10.1021/ol802343z
