# Supplementary material for: Revisiting the bromination of 3β-hydroxycholest-5-ene with CBr4/PPh3 and the subsequent azidolysis of the resulting bromide, disparity in stereochemical behavior
Source: Beilstein J Org Chem. 2023 Jan 27;19:91–9. doi: 10.3762/bjoc.19.9 (PMC9887750; doi:10.3762/bjoc.19.9)

# checkCIF/PLATON report

You have not supplied any structure factors. As a result the full set of tests cannot be run.

THIS REPORT IS FOR GUIDANCE ONLY. IF USED AS PART OF A REVIEW PROCEDURE FOR PUBLICATION, IT SHOULD NOT REPLACE THE EXPERTISE OF AN EXPERIENCED CRYSTALLOGRAPHIC REFEREE.

No syntax errors found.    CIF dictionary    Interpreting this report

## Datablock: 2

---

```
Bond precision:   C-C = 0.0040 A          Wavelength=0.71073

Cell:               a=7.5850(2)       b=15.9238(4)       c=19.4795(4)
                    alpha=90          beta=90            gamma=90
Temperature:        170 K

                 Calculated                   Reported
Volume           2352.77(10)                  2352.77(10)
Space group      P 21 21 21                   P 21 21 21
Hall group       P 2ac 2ab                    P 2ac 2ab
Moiety formula   C27 H44                      C27 H44
Sum formula      C27 H44                      C27 H44
Mr               368.62                       368.62
Dx,g cm-3        1.041                        1.041
Z                4                            4
Mu (mm-1)        0.057                        0.057
F000             824.0                        824.0
F000'            824.26
h,k,lmax         9,20,24                      9,20,24
Nref             5200[ 2959]                  5169
Tmin,Tmax        0.980,0.986                  0.514,0.746
Tmin'            0.977

Correction method= # Reported T Limits: Tmin=0.514 Tmax=0.746
AbsCorr = MULTI-SCAN

Data completeness= 1.75/0.99        Theta(max)= 27.101

                                                    wR2(reflections)=
R(reflections)= 0.0531( 3861)                       0.1314( 5169)

S = 1.035                  Npar= 249
```

The following ALERTS were generated. Each ALERT has the format
**test-name_ALERT_alert-type_alert-level.**
Click on the hyperlinks for more details of the test.

## Alert level G

```
PLAT791_ALERT_4_G Model has Chirality at C8          (Sohnke SpGr)          S Verify
PLAT791_ALERT_4_G Model has Chirality at C9          (Sohnke SpGr)          S Verify
PLAT791_ALERT_4_G Model has Chirality at C10         (Sohnke SpGr)          R Verify
PLAT791_ALERT_4_G Model has Chirality at C13         (Sohnke SpGr)          S Verify
PLAT791_ALERT_4_G Model has Chirality at C14         (Sohnke SpGr)          S Verify
PLAT791_ALERT_4_G Model has Chirality at C17         (Sohnke SpGr)          R Verify
PLAT791_ALERT_4_G Model has Chirality at C20         (Sohnke SpGr)          R Verify
```

0 **ALERT level A** = Most likely a serious problem - resolve or explain
0 **ALERT level B** = A potentially serious problem, consider carefully
0 **ALERT level C** = Check. Ensure it is not caused by an omission or oversight
7 **ALERT level G** = General information/check it is not something unexpected

0 ALERT type 1 CIF construction/syntax error, inconsistent or missing data
0 ALERT type 2 Indicator that the structure model may be wrong or deficient
0 ALERT type 3 Indicator that the structure quality may be low
7 ALERT type 4 Improvement, methodology, query or suggestion
0 ALERT type 5 Informative message, check

It is advisable to attempt to resolve as many as possible of the alerts in all categories. Often the minor alerts point to easily fixed oversights, errors and omissions in your CIF or refinement strategy, so attention to these fine details can be worthwhile. In order to resolve some of the more serious problems it may be necessary to carry out additional measurements or structure refinements. However, the purpose of your study may justify the reported deviations and the more serious of these should normally be commented upon in the discussion or experimental section of a paper or in the "special_details" fields of the CIF. checkCIF was carefully designed to identify outliers and unusual parameters, but every test has its limitations and alerts that are not important in a particular case may appear. Conversely, the absence of alerts does not guarantee there are no aspects of the results needing attention. It is up to the individual to critically assess their own results and, if necessary, seek expert advice.

**Publication of your CIF in IUCr journals**

A basic structural check has been run on your CIF. These basic checks will be run on all CIFs submitted for publication in IUCr journals (*Acta Crystallographica*, *Journal of Applied Crystallography*, *Journal of Synchrotron Radiation*); however, if you intend to submit to *Acta Crystallographica Section C* or *E* or *IUCrData*, you should make sure that full publication checks are run on the final version of your CIF prior to submission.

**Publication of your CIF in other journals**

Please refer to the *Notes for Authors* of the relevant journal for any special instructions relating to CIF submission.

---

**PLATON version of 18/05/2022; check.def file version of 17/05/2022**

**Datablock 2** - ellipsoid plot

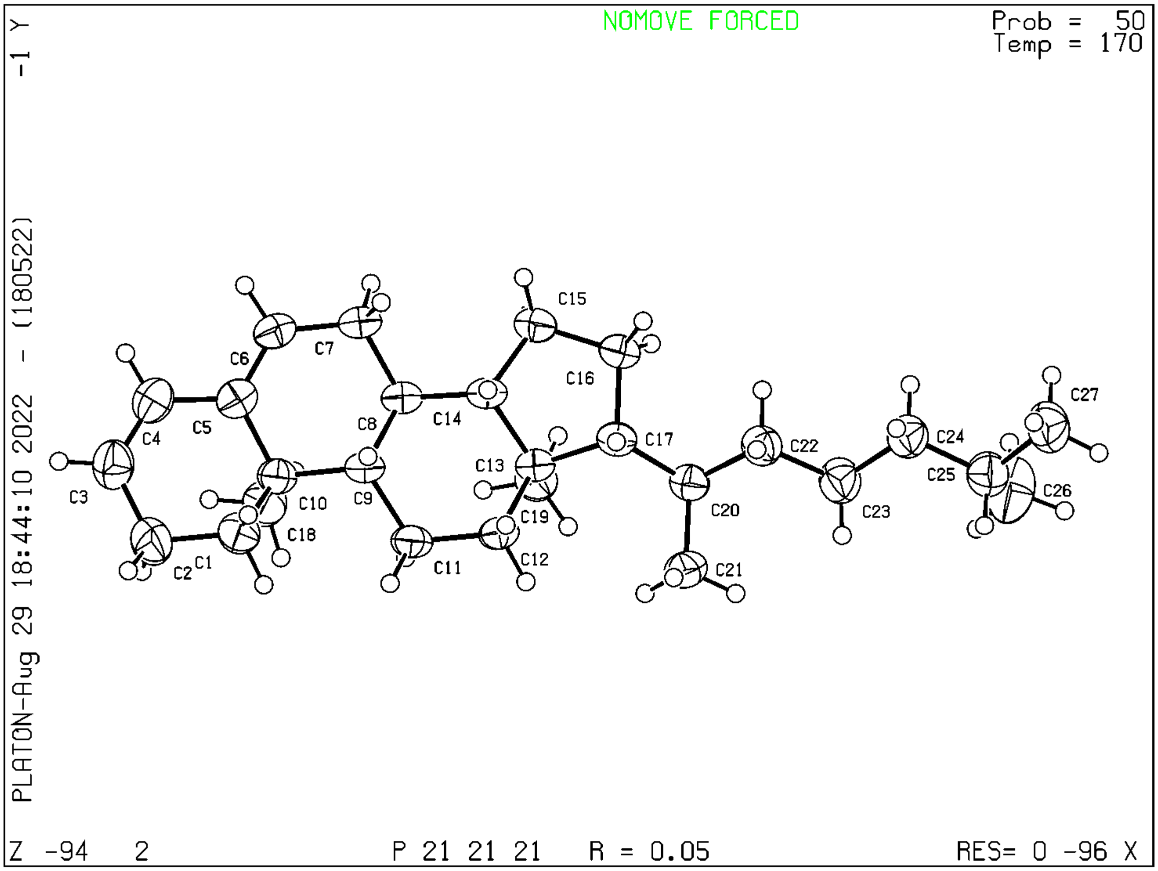

Supplement: File 2 — Crystallographic information files for compounds 4 and 9. [file Beilstein_J_Org_Chem-19-91-s002.zip › 9+Checkcif+Report.pdf]
