# Supplementary material for: Revisiting the bromination of 3β-hydroxycholest-5-ene with CBr4/PPh3 and the subsequent azidolysis of the resulting bromide, disparity in stereochemical behavior
Source: Beilstein J Org Chem. 2023 Jan 27;19:91–9. doi: 10.3762/bjoc.19.9 (PMC9887750; doi:10.3762/bjoc.19.9)

checkCIF/PLATON report

You have not supplied any structure factors. As a result the full set of tests cannot be run.

THIS REPORT IS FOR GUIDANCE ONLY. IF USED AS PART OF A REVIEW PROCEDURE FOR PUBLICATION, IT SHOULD NOT REPLACE THE EXPERTISE OF AN EXPERIENCED CRYSTALLOGRAPHIC REFEREE.

No syntax errors found.   CIF dictionary   Interpreting this report

Datablock: 3

```
Bond precision:   C-C = 0.0168 A           Wavelength=0.71073

Cell:             a=11.4127(12)     b=7.5896(9)        c=28.603(5)
                  alpha=90          beta=90.077(13)    gamma=90
Temperature:      120 K

                 Calculated                  Reported
Volume           2477.5(6)                   2477.5(6)
Space group      P 21                        P 1 21 1
Hall group       P 2yb                       P 2yb
Moiety formula   C27 H45 Br                  C27 H45 Br
Sum formula      C27 H45 Br                  C27 H45 Br
Mr               449.53                      449.54
Dx,g cm-3        1.205                       1.205
Z                4                           4
Mu (mm-1)        1.669                       1.669
F000             968.0                       968.0
F000'            967.13
h,k,lmax         15,10,39                    15,10,37
Nref             13372[ 7169]                11562
Tmin,Tmax        0.721,0.911                 0.544,1.000
Tmin'            0.651

Correction method= # Reported T Limits: Tmin=0.544 Tmax=1.000
AbsCorr = GAUSSIAN

Data completeness= 1.61/0.86        Theta(max)= 29.160

R(reflections)= 0.0982( 5812)                        wR2(reflections)=
                                                     0.3085( 11562)
S = 1.055                   Npar= 467
```

The following ALERTS were generated. Each ALERT has the format
test-name_ALERT_alert-type_alert-level.
Click on the hyperlinks for more details of the test.

Alert level B

```
PLAT110_ALERT_2_B ADDSYM Detects Potential Lattice Translation ...          ? Check
```

Author Response: The unit cell is extremely close to higher (orthorhombic) symmetry. which was thoroughly explored. However, close examination of the metrics confirm that the assignment as monoclinic to be correct.

```
PLAT341_ALERT_3_B Low Bond Precision on  C-C Bonds ..............    0.01683 Ang.
```

Author Response: The crystals supplied were all heavily twinned, with data being collected on the best of those tested. The twinning could not be accounted for by a suitable twin law, and was therefore left untreated in the final model, which had a detrimental effect on the bond precision of the light atoms. However, the identity of the structure was clearly established without issue.

Alert level C

```
PLAT084_ALERT_3_C High wR2 Value (i.e. > 0.25) ..................       0.31 Report
PLAT213_ALERT_2_C Atom C54             has ADP max/min Ratio .....       3.1 prolat
PLAT213_ALERT_2_C Atom C55             has ADP max/min Ratio .....       3.1 prolat
PLAT230_ALERT_2_C Hirshfeld Test Diff for    C75      --C77      .        5.4 s.u.
PLAT242_ALERT_2_C Low    'MainMol' Ueq as Compared to Neighbors of        C75 Check
```

Alert level G

```
PLAT003_ALERT_2_G Number of Uiso or Uij Restrained non-H Atoms ...         18 Report
PLAT072_ALERT_2_G SHELXL First  Parameter in WGHT  Unusually Large       0.12 Report
PLAT112_ALERT_2_G ADDSYM Detects New (Pseudo) Symm. Elem         B        100 %Fit
PLAT113_ALERT_2_G ADDSYM Suggests Possible Pseudo/New  Space Group        P21 Check
             Note: (Pseudo) Lattice Translation Implemented
PLAT171_ALERT_4_G The CIF-Embedded .res File Contains EADP Records          5 Report
PLAT187_ALERT_4_G The CIF-Embedded .res File Contains RIGU Records          2 Report
PLAT791_ALERT_4_G Model has Chirality at C3         (Sohnke SpGr)           S Verify
PLAT791_ALERT_4_G Model has Chirality at C8         (Sohnke SpGr)           S Verify
PLAT791_ALERT_4_G Model has Chirality at C9         (Sohnke SpGr)           S Verify
PLAT791_ALERT_4_G Model has Chirality at C10        (Sohnke SpGr)           R Verify
PLAT791_ALERT_4_G Model has Chirality at C13        (Sohnke SpGr)           S Verify
PLAT791_ALERT_4_G Model has Chirality at C14        (Sohnke SpGr)           S Verify
PLAT791_ALERT_4_G Model has Chirality at C17        (Sohnke SpGr)           R Verify
PLAT791_ALERT_4_G Model has Chirality at C20        (Sohnke SpGr)           R Verify
PLAT791_ALERT_4_G Model has Chirality at C53        (Sohnke SpGr)           S Verify
PLAT791_ALERT_4_G Model has Chirality at C58        (Sohnke SpGr)           S Verify
PLAT791_ALERT_4_G Model has Chirality at C59        (Sohnke SpGr)           S Verify
PLAT791_ALERT_4_G Model has Chirality at C60        (Sohnke SpGr)           R Verify
```

```
PLAT791_ALERT_4_G Model has Chirality at C63         (Sohnke SpGr)          S Verify
PLAT791_ALERT_4_G Model has Chirality at C64         (Sohnke SpGr)          S Verify
PLAT791_ALERT_4_G Model has Chirality at C67         (Sohnke SpGr)          R Verify
PLAT791_ALERT_4_G Model has Chirality at C70         (Sohnke SpGr)          R Verify
PLAT860_ALERT_3_G Number of Least-Squares Restraints ............         130 Note
PLAT941_ALERT_3_G Average HKL Measurement Multiplicity ..........         4.9 Low
PLAT952_ALERT_5_G Calculated (ThMax) and CIF-Reported Lmax Differ.          2 Units
```

```
   0 ALERT level A = Most likely a serious problem - resolve or explain
   2 ALERT level B = A potentially serious problem, consider carefully
   5 ALERT level C = Check. Ensure it is not caused by an omission or oversight
  25 ALERT level G = General information/check it is not something unexpected

   0 ALERT type 1 CIF construction/syntax error, inconsistent or missing data
   9 ALERT type 2 Indicator that the structure model may be wrong or deficient
   4 ALERT type 3 Indicator that the structure quality may be low
  18 ALERT type 4 Improvement, methodology, query or suggestion
   1 ALERT type 5 Informative message, check
```

It is advisable to attempt to resolve as many as possible of the alerts in all categories. Often the minor alerts point to easily fixed oversights, errors and omissions in your CIF or refinement strategy, so attention to these fine details can be worthwhile. In order to resolve some of the more serious problems it may be necessary to carry out additional measurements or structure refinements. However, the purpose of your study may justify the reported deviations and the more serious of these should normally be commented upon in the discussion or experimental section of a paper or in the "special_details" fields of the CIF. checkCIF was carefully designed to identify outliers and unusual parameters, but every test has its limitations and alerts that are not important in a particular case may appear. Conversely, the absence of alerts does not guarantee there are no aspects of the results needing attention. It is up to the individual to critically assess their own results and, if necessary, seek expert advice.

Publication of your CIF in IUCr journals

A basic structural check has been run on your CIF. These basic checks will be run on all CIFs submitted for publication in IUCr journals (*Acta Crystallographica*, *Journal of Applied Crystallography*, *Journal of Synchrotron Radiation*); however, if you intend to submit to *Acta Crystallographica Section C* or *E* or *IUCrData*, you should make sure that full publication checks are run on the final version of your CIF prior to submission.

Publication of your CIF in other journals

Please refer to the *Notes for Authors* of the relevant journal for any special instructions relating to CIF submission.

PLATON version of 18/05/2022; check.def file version of 17/05/2022

Datablock 3 - ellipsoid plot

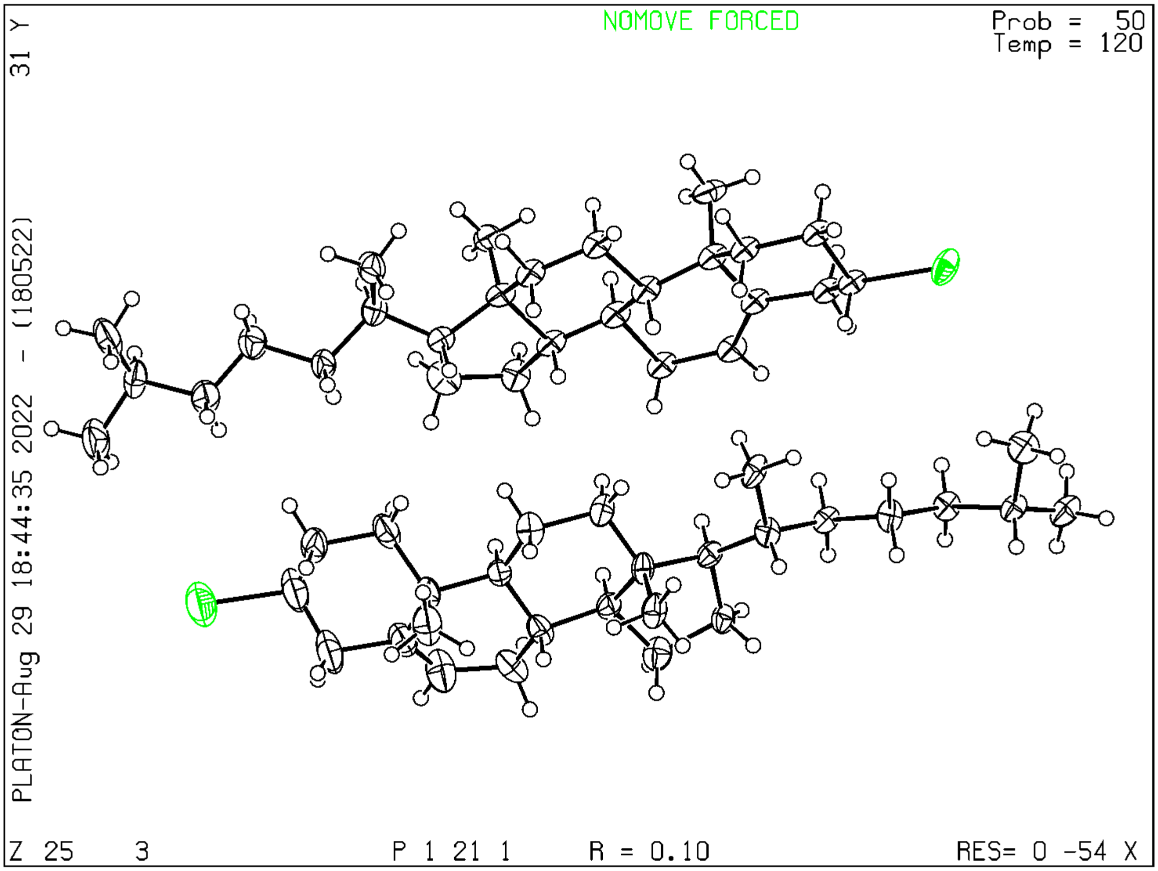

Supplement: File 2 — Crystallographic information files for compounds 4 and 9. [file 4+Checkcif+Report.pdf › 4+Checkcif+Report.pdf]
